# Disrupting the vicious cycle of malnutrition and enteropathy: advancing strategies to improve child physical and neurocognitive outcomes

**DOI:** 10.3389/fpubh.2026.1792466

**Published:** 2026-09-01

**Authors:** M. Khalid Ijaz, Raymond W. Nims, Joseph R. Rubino, Bushra S. F. Hasan, Maryum K. Ijaz, Frank Y. Xu, Charles P. Gerba

**Affiliations:** 1 Global Research & Development for Lysol and Dettol, Reckitt Benckiser LLC, Montvale, NJ, United States; 2 Syner-G Biopharma, Boulder, CO, United States; 3 Home and Community Hygiene, Parsippany, NJ, United States; 4 Water-WISER CDT, University of Leeds, Leeds, United Kingdom; 5 Department of Psychiatry & Behavioral Sciences, Tulane University School of Medicine, New Orleans, LA, United States; 6 Water & Energy Sustainable Technology Center, University of Arizona, Tucson, AZ, United States

**Keywords:** baby WASH, chronic childhood malnutrition, environmental enteropathy, malnutrition and infection cycle, non-pharmaceutical interventions, recurring enteric infections, targeted household hygiene, water sanitation and hygiene (WASH)

## Abstract

**Background:**

Recognition of the synergistic cycle of malnutrition, immune dysfunction, and recurrent enteric infection in children and its role in linear growth stunting and impaired cognitive development (the “stunting syndrome”), dates back to the 1960s. Early hypotheses emphasized reduced intestinal absorption associated with diarrhea as the primary enteropathy driving malnutrition and developmental deficits. However, recent evidence indicates that a subclinical condition termed environmental enteropathy (EE), or environmental enteric dysfunction (EED), may be a more critical contributor. Mitigating the adverse impacts of EE/EED requires a holistic, integrated approach that combines improved nutrition, water, sanitation, and hygiene (WASH), appropriate vaccination, and targeted non-pharmaceutical hygiene interventions designed to prevent recurrent enteric infections. These interventions aim to reduce pathogen exposure in infants’ immediate environments, a prerequisite for achieving sustained physical and neurocognitive development and, ultimately, economic progress in low- and middle-income countries (LMICs).

**Objective:**

This Hypothesis and Theory article synthesizes current understanding of EE/EED as a proximate driver of chronic childhood malnutrition and its major sequelae: stunting, impaired neurocognitive development, immune dysfunction (including poor pediatric vaccine response), and nutrient malabsorption. We hypothesize the necessity of reducing enteric infection occurrence as a cornerstone for breaking the malnutrition–infection cycle and propose that non-pharmaceutical interventions targeting pathogen exposure in household and infant play environments are essential. In addition to pathogen exposure, we examine other determinants of malnutrition, including microbiome dysbiosis and epigenetic alterations as possible targets for therapeutic interventions intended to mitigate negative outcomes on health and nutrition.

**Methods:**

We reviewed literature addressing malnutrition, immune impacts, enteric infections, EE, growth stunting, cognitive impairment, epigenetic alterations, and mitigation strategies including WASH, baby WASH, hand and surface hygiene, and vaccination, using databases such as PubMed, Embase, and Google Scholar.

**Results:**

The synergistic malnutrition and infection cycle underlying the stunting syndrome comprises multiple interdependent components: chronic malnutrition, immune dysfunction, recurrent pathogen assault, microbiome dysbiosis, and EE/EED. Previous interventions have largely failed, likely because they targeted isolated components rather than the entire cycle. Here, we describe this cycle, its interactions, and prior attempts at disruption. We advocate for a novel, integrated approach, termed *baby WASH* or environmental hygiene, focused on creating hygienically clean environments where infants live and play, thereby reducing exposure to enteric pathogens. We also explore the link between malnutrition, EE/EED, and epigenetic reprogramming, as a contributor to health risks. Importantly, malnutrition is linked to epigenetic reprogramming, which contributes to neurocognitive impairment. We hypothesize that by combining baby WASH strategies with emerging pharmaceutical interventions addressing epigenetic and nutritional deficits, and associated socioeconomic and political factors, a comprehensive maternal–child health framework may be established to interrupt this vicious cycle.

**Conclusion:**

Preventing recurrent pathogen exposure during both prenatal and postnatal periods is hypothesized to be essential to interrupting the malnutrition and infection cycle and reducing stunting. Interventions must address infants’ immediate living, feeding, and educational environments, as well as maternal environments during gestation. Early-life nutritional deficiencies may influence epigenetic programming, with developmental consequences. Breaking this cycle remains an unmet global health priority, warranting urgent international attention due to the human and economic tolls in resource-limited settings LMICs.

## 1 Introduction

Awareness of the importance of the so-called *vicious synergistic cycle of malnutrition, immune dysfunction, and enteric infection* (synergistic malnutrition and infection cycle) in young children for causing decreased linear growth and impacts on cognitive development began in the 1960s (1–6). Initially, it was believed that reduced intestinal absorption associated with diarrhea (and the types of enteric infections leading to diarrhea) represented the most important enteropathy leading to malnutrition and the associated developmental deficits (1, 2, 6–14). Therefore, it was believed that efforts to reduce the frequency of infections causing diarrhea, together with improved dietary status, might reduce the adverse developmental impacts of the malnutrition cycle [impacts on linear growth and cognitive development, collectively referred to as the stunting syndrome (15)]. These expectations have not been borne out, as nutritional interventions alone have not displayed the expected mitigational impacts on stunting (15), and the reported associations between the stunting syndrome and occurrence of diarrhea have been relatively weak (14, 15).

The association between recurring enteric infections and malnutrition has led to a variety of clinical trials investigating the potential beneficial impacts of improved water quality, household and community sanitation (primarily involving the safe disposal of human excreta), and personal sanitation (hand washing with soap), collectively termed WASH interventions, on the occurrence of childhood stunting (15–19). Studies attempting to determine the beneficial impacts of WASH interventions targeting prevention of enteric infections on measures of linear growth have reported varying results, with the most typical results showing some impact, though perhaps not the degree of impact expected (19–23), likely due in part to inconsistences in the reporting (24).

A breakthrough in this field occurred when various investigators realized that a subclinical syndrome termed environmental enteropathy (EE), also termed environmental enteric dysfunction (EED), appeared to be more important in the context of childhood stunting than diarrhea (25–37). Stated another way, diarrheagenic and non-diarrheagenic enteric infections leading to a variety of enteric structural and functional impacts appeared to be more important in the causation of stunting than diarrhea itself. On a high level, EE/EED may be characterized (25, 30, 33–35, 37) as a combination of structural changes, including blunting (decreased height) of small intestinal villi and increased depth of small intestinal crypts (crypt hyperplasia), and indications of inflammation (lymphocytic infiltration of the lamina propria and increases in intraepithelial lymphocytes). Functional changes include adverse impacts on the absorptive function of the small intestine, increased intestinal permeability, and inappropriate engagement of the innate and adaptive immune responses of the intestines (i.e., a chronic hyperimmune state). Translocation of bacteria or bacterial bioproducts from the intestinal lumen into the systemic circulation may occur due to the decreased barrier function of the intestinal cell tight junctions and likely contributes to the hyperimmune state associated with EE (25, 30, 33, 35, 37).

Another sequella of (or contributor to) EE/EED is microbiome dysbiosis (also referred to as the *pathobiome*; that is characterized by reduced diversity of the enteric microbiome, with decrease in taxa associated with healthy childhood growth and shifting to more pathogenic taxa) (30, 38). The changes in intestinal absorption and permeability associated with EE/EED may be monitored by measuring the ratio of lactulose to mannitol in the urine, with higher ratios being indicative of increased permeability and malabsorption. Other biomarkers of EE/EED have been characterized (30, 39, 40), and the transcriptomes of the impacted small intestinal cells have been characterized (34, 35). Recent preclinical and clinical studies have identified the critical role of the early-life gut microbiome in shaping physiological systems, including metabolism, immune development, intestinal function, and hormonal regulation (41–44). While bacteria have traditionally been the primary focus in microbiome research, emerging evidence suggests that viruses and fungi also contribute significantly to these processes. Disruptions in the balance and composition of these microbial communities can trigger pathophysiological pathways that impair nutrient absorption, immune homeostasis, and endocrine signaling, ultimately contributing to the impacts of child undernutrition (41–44).

Unfortunately, it appears that addressing childhood stunting is not as simple as mitigating the causes of diarrhea, and not as simple as improving nutritional quantity and quality, vaccination programs, water quality, community sanitation practices, or implementing hand washing. These approaches are important and necessary interventions, but something more holistic and comprehensive may be needed. Otherwise, administration of nutrition and vaccines will fail, just as certainly as will adding water to a *leaky bucket* (4). The description of the malnutrition cycle should therefore be extended to include EE/EED, as in *chronic childhood malnutrition, immune dysfunction, enteric pathogen assault, and EE/EED*. We have aligned with this more complete version of the malnutrition and infection cycle which accounts for the importance of EE/EED and the factors that ultimately lead to this enteropathy, subsequently resulting in the stunting syndrome. We also advocate for additional approaches for mitigating stunting which have been suggested previously but not fully articulated nor evaluated for relevance and impact in field trials.

## 2 Methods

**Figure 1:**
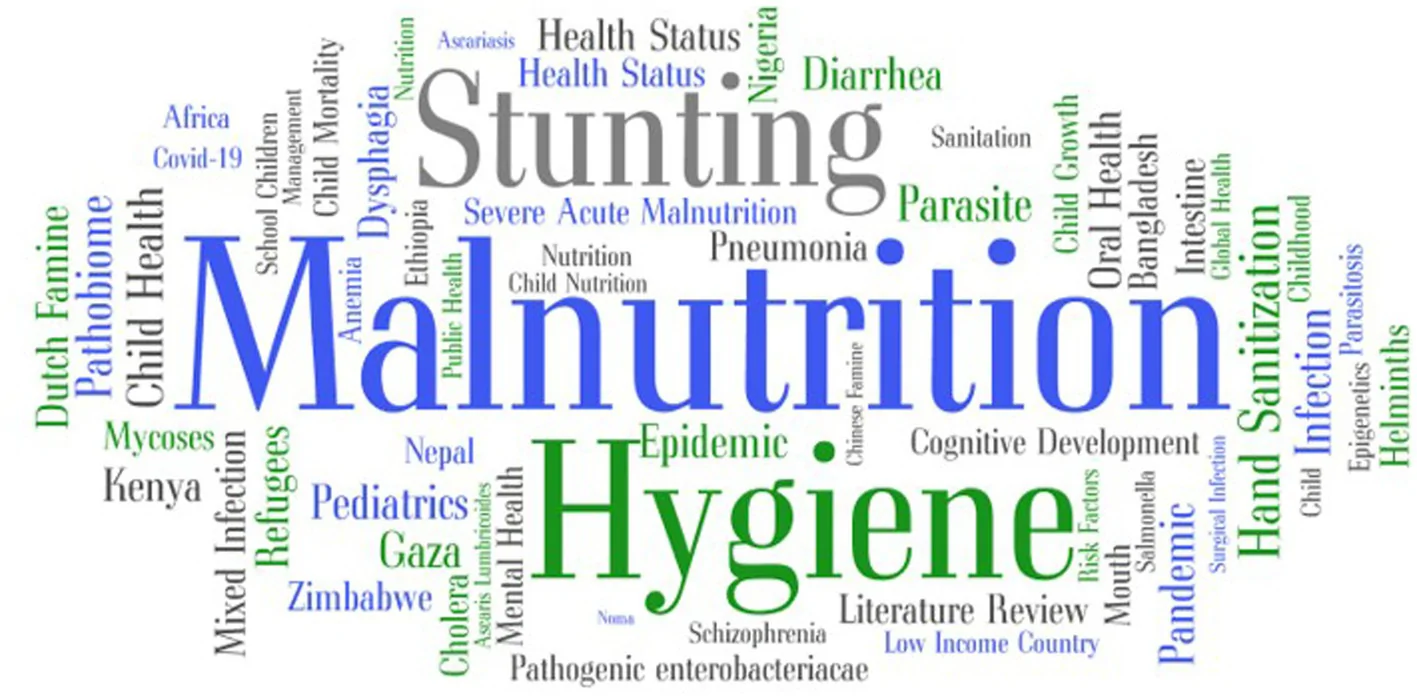
Word cloud visualization of enteric pathogens, malnutrition, stunting, environmental enteropathy and hygiene, in all subject areas. Data source Scopus. Relevance of keyphrase growing (AAA) or declining (AAA).

This narrative review was designed to gather and evaluate information in the primary scientific literature on the synergism between malnutrition, hand and surface hygiene, and EE/EED, as these impact childhood physical and cognitive development (see Figure 1). Additional factors requiring further investigation were also examined, with the goal of advancing a more comprehensive approach to therapeutic and non-therapeutic interventions. The databases searched included Embase, PubMed, SciVal, Google, and Google Scholar. The search strings employed have been displayed in Supplementary Tables S1–S5. The intent was not to systematically review these topics, as numerous reviews have been published on the topics of childhood stunting, malnutrition and its impacts on the immune system and infection prevalence, and on EE/EED, and it is not our intention to reproduce or improve on these. Rather, we have drawn, from these sources, information required to convey, at a high level, information on the components of the malnutrition and infection cycle and to establish our hypothesis regarding gaps in current strategies in addressing the stunting syndrome and its proximate cause, EE/EED.

## 3 Results

As a starting point in this Hypothesis and Theory article, we describe the cycle of events currently believed to underlie the stunting syndrome. As mentioned above, we believe that this cycle is best described as a cycle of *chronic childhood malnutrition, immune dysfunction, enteric pathogen assault*, and EE/EED. This malnutrition and infection cycle is described in the next section. The following sections of the paper deal with the individual aspects (components) of the cycle. Finally, we discuss interventions for mitigating stunting that have been considered and evaluated in clinical (field) trials and advocate for additional interventions that should be evaluated.

### 3.1 The synergistic cycle of chronic childhood malnutrition, immune dysfunction, enteric pathogen assault, and environmental enteropathy

**Figure 2:**
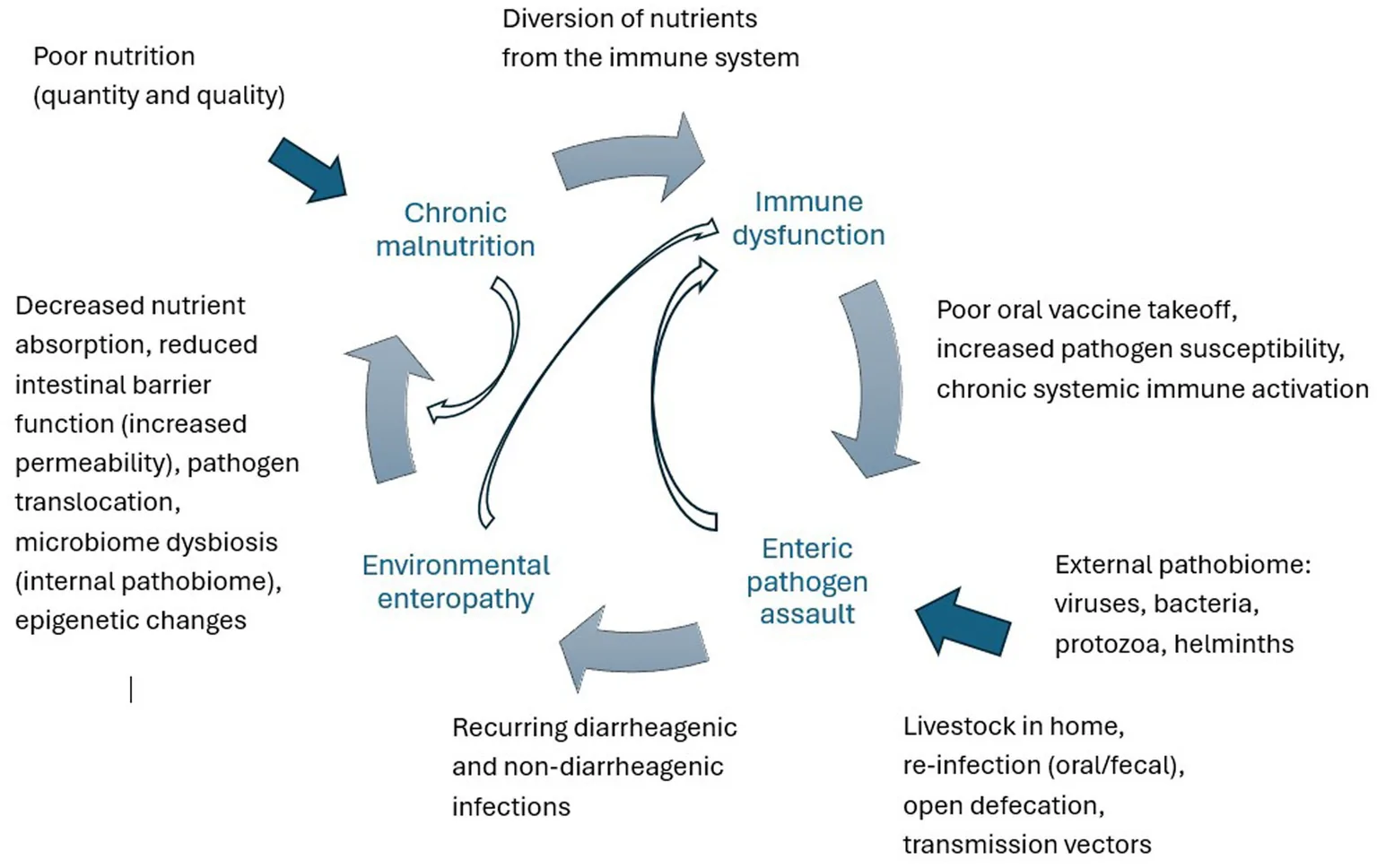
Schematic depiction of the synergistic cycle of chronic childhood malnutrition, immune dysfunction, enteric pathogen assault, and environmental enteropathy.

The cycle of events displayed in Figure 2 is intended to provide an overview of the events thought to lead to the stunting syndrome based on current thinking (25–30) as well as leveraging previous schematic renderings of the malnutrition cycle (10–12, 32, 33). As with any cyclic phenomenon, it is difficult to establish a starting point, and Figure 2 is somewhat simplistic in that deviations from the sequence of events depicted may occur, as shown in the figure. In particular, the establishment of immune dysfunction may represent a sequella of nutrient deficiency, enteric pathogen assault, and EE/EED. We take as a logical starting point chronic childhood malnutrition, and it is important to realize that malnutrition typically begins in the womb of the mother experiencing poor nutrition prior to and during gestation and extends into the post-natal period. This then contributes to the manifestations of immune dysfunction which will be described in more detail in a subsequent section of the paper. The immune dysfunction involves chronic systemic and intestinal immune activation and inflammation and is thought to contribute to poor responsiveness to vaccination and increased susceptibility to enteric and other pathogens. Recurring assaults with enteric pathogens (viruses, bacteria, protozoans, and helminths) occur because of increased pathogen susceptibility and as a consequence of living in a pathobiome-dominated environment. This pathogen assault is thought to drive a shift in the intestinal microbiome toward an internal pathobiome, which likely represents the proximate cause of EE/EED. Environmental enteropathy, in turn, induces structural and functional damage to the small intestine, exacerbating nutrient malabsorption and worsening malnutrition. The malnutrition cycle (Figure 2) is highly resilient, with each step reinforcing the next, making interruption of the cycle extremely challenging. Interventions that target only isolated components of the cycle are unlikely to succeed; all contributing factors must be addressed comprehensively, much like plugging every leak in the bucket must be done for it to hold water.

### 3.2 Chronic malnutrition, a starting point in the synergistic malnutrition and infection cycle

The link between childhood infection and severe malnutrition was established in the late 1950s by Scrimshaw and coworkers (45, 46). By this time, it was already clear that growth stunting was related to malnutrition (Figure 2), which becomes especially severe after weaning. In keeping with this finding, studies have demonstrated that the prevalence of childhood stunting is low soon after birth but increases with age, reaching a maximum prevalence by 20 months of age (9). The possible beneficial impact of supplemental nutrition on childhood illness frequency was suggested in this early work (45), although in the intervening years it has become clear that simply improving nutritional intake does not represent an effective intervention for interrupting the synergistic cycle of malnutrition and infection, and the ensuing stunting syndrome (4). By the late 1960s, it was realized that malnutrition and infection are synergistic, in that infections result in malnutrition and malnutrition predisposes to further infections, resulting in the combined impacts of malnutrition and infection on outcomes such as kwashiorkor being greater than those of either factor alone (2, 45). Kwashiorkor is a clinical syndrome characterized by a weight-to-height ratio or body mass index less than 70% and pitting edema in the feet (5).

While earlier work (4, 45) emphasized protein/energy malnutrition especially, it has subsequently become clear that the impacts of malnutrition not only are due to deficiencies in proteins, but also in lipids and carbohydrates (and energy or calories), specific amino acids, vitamins, and minerals (2). These deficiencies have been noted to be sequellae of certain infections (e.g., viral, bacterial, protozoan, and helminth), and adverse impacts of the nutritional deficiencies on the immune system have been noted (2).

**Figure 3:**
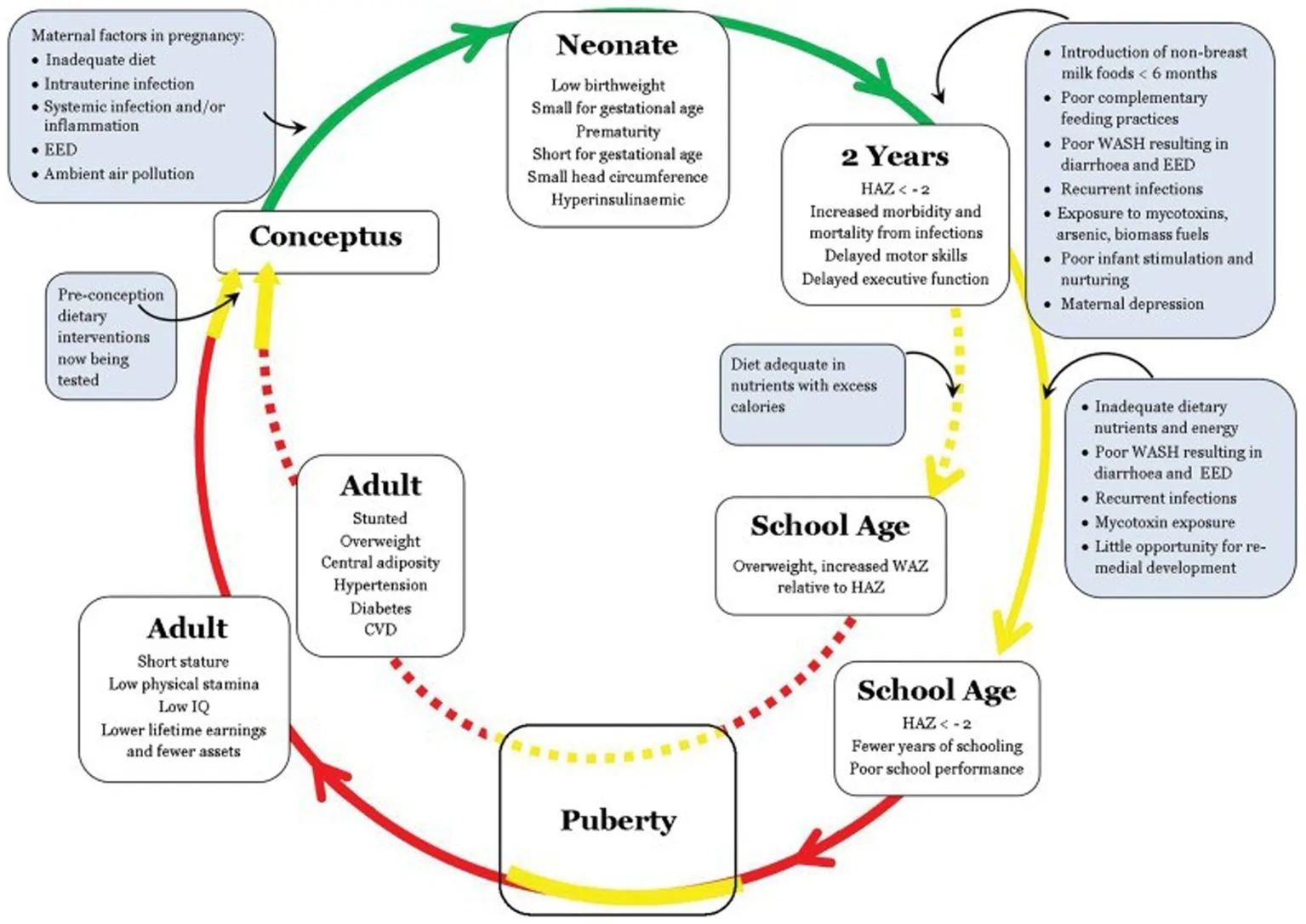
The stunting syndrome. The green pathway denotes the period between conception and 2 years (‘the first 1,000 days’) when stunting and probably all associated pathology are most responsive to, or preventable by, interventions. The yellow pathway denotes periods between age 2 years and mid-childhood and during the adolescent growth spurt when some catch-up in linear growth may occur, though effects during these periods on other components of the stunting syndrome (e.g., cognition and immune function) are less clear. The short yellow pathway before Conceptus reflects evidence that dietary interventions targeting stunted women during the pre-conception period improve birth outcomes. The red pathway denotes periods when the stunting syndrome appears unresponsive to interventions. Blue boxes list age-specific causative or aggravating factors. White boxes describe common age-specific outcomes. Between 2 years and adulthood, the pathways diverge to denote: Dashed line, a stunted child whose environment becomes more affluent with abundant access to food, causing excessive weight gain; Solid line: A stunted child whose environment remains resource-constrained/food insecure. From (15) per Creative Commons Attribution Non-Commercial License 3.0. EED, environmental enteric dysfunction; HAZ, height-for-age Z-score; IQ, intelligence quotient; WASH, improved water, sanitation, and hygiene; WAZ, weight-for-age Z-score.

There are a variety of factors which contribute to childhood malnutrition, which occurs primarily in developing countries but may also be found in developed countries (4). The factors center on inadequate household food security (availability and control of food such that there is access for all family members to enough food for an active and healthy life) and go beyond simple poverty to include also political and governmental policy factors (7). There appears to be a consensus that malnutrition is a primary proximate cause of childhood stunting, where stunting is used in the broader sense to include adverse impacts both on linear growth and cognitive development (10, 15, 17, 47, 48). The key factors and chronologies are displayed in Figure 3 [reproduced from Prendergast and Humphrey (15)] and reflect the importance of influences occurring during gestation and during the first 2 years of neonatal life. Of course, it is difficult to dissect out the impacts of malnutrition from those of recurrent enteric infection and adverse impacts on the immune system (and the subsequent EE/EED), as will become clearer as we proceed with our description of the synergistic malnutrition and infection cycle.

The multifactorial causes of childhood malnutrition, particularly in low- and middle-income countries, include the profound effects of armed conflict and the use of starvation as a weapon of war. The Dutch famine, or “Hunger Winter” of 1944–1945 in German-occupied Netherlands, provides a striking historical example of famine-induced developmental health effects (49). Prenatal exposure to famine, especially during early gestation, has been found to be associated with increased risk of neuropsychiatric disorders, including anxiety, depression, and cognitive impairment persisting into midlife. Those exposed to famine in mid to late gestation were, overall, shorter and thinner, with smaller head circumferences. These findings align with the well-established principle that early gestation represents a critical window in organogenesis, during which environmental insults exert the most extensive and enduring physiological consequences (49).

Between 1959 and 1961, China experienced a devastating famine that resulted in an estimated 20 to 30 million excess deaths, along with widespread miscarriages and stillbirths. Individuals born during this period were exposed to severe nutritional deprivation in utero and early life, leading to health consequences (50). The Gaza Strip represents a contemporary example of conflict-driven malnutrition, where the collapse of food systems, healthcare, and WASH infrastructure has led to widespread acute malnutrition in children and conditions that are expected to promote EE/EED through recurrent infection, microbiome disruption, and impaired intestinal barrier function. Unfortunately, these pathophysiological impacts relating to stunting may take years to develop, and the stunting that is expected will only be evident in years to come (51, 52).

### 3.3 Epigenetic changes associated with childhood malnutrition

As depicted in Figure 2, malnutrition can cause impacts on childhood development. One mechanism for these impacts of malnutrition appears to be epigenetic changes impacting linear growth and neurocognitive development. Epigenetics refers to heritable changes in gene function that occur in the absence of alterations to the primary DNA sequence of the gene. These changes may modify fetal epigenetic programming (50, 53–64). Key epigenetic mechanisms (Table 1) include DNA methylation, histone modification, and non-coding RNA regulation. Pathogen infections exert significant influences over epigenetic regulation in the gastrointestinal tract, inducing alterations in DNA methylation, histone post-translational modifications, and non-coding RNA expression. These epigenetic effects are largely mediated by pathogen-derived metabolites, including short-chain fatty acids (SCFAs), polyamines, and vitamins, which interact with host chromatin-modifying enzymes and metabolic pathways. Disruption of microbial signaling can compromise one-carbon metabolism, reducing the pool of methyl donors required for DNA methylation and thereby reprogramming host gene expression (55). This epigenetic reconfiguration contributes to dysregulated transcriptional networks associated with inflammatory bowel disease, colorectal cancer, and other inflammation-driven gastrointestinal disorders (56). The effects of the Dutch famine (49) and the Chinese famine (50) were found to include altered methylation of the insulin receptor (*INSR*) gene and the insulin-like growth factor 2 (*IGF2*) gene, a key regulator of tyrosine hydroxylase, an enzyme involved in dopamine synthesis. This illustrates how early-life nutritional deprivation can disrupt dopaminergic pathways.

**Table 1:** Epigenetic mechanisms influenced by malnutrition and subsequent outcomes.

Step	Mechanism/effect	Example/outcome	References
Nutrient deficiency	Decrease in methyl donors (methionine, folate, etc.)	Impaired one-carbon metabolism	Kadayifci et al. (57); Anderson et al. (58); Feinberg(59)
DNA methylation	Global/locus-specific hypomethylation	Altered metabolic/immune gene expression	Feinberg(59); Schulze et al. (60); Lee et al. (61)
Histone modification	Redistribution of H3K4me3 markers	Changes in chromatin, gene regulation	Uchiyama et al. (62)
Gene expression	Dysregulation of growth/metabolic/immunity genes	Stunting, metabolic syndrome	Gomez-Verjan et al. (50); Schulze et al. (60); Wells et al. (63); Campisano et al. (64)

### 3.4 Immune dysfunction, a sequella and a contributor to the synergistic malnutrition and infection cycle

How do chronic malnutrition and the other components of the synergistic malnutrition and infection cycle (Figure 2) lead to immune dysfunction, and what is the nature of this dysfunction? The effects of malnutrition itself on the immune system stem from insufficiencies in nutrients necessary for function. For instance, direct effects of malnutrition on antibody generation, leukocyte function, complement formation, and cytokine generation, have been described (2, 4, 5, 43) and have been attributed to deficiencies in protein, specific amino acids, vitamins, and minerals (2).

The recurring pathogen assault (from the external pathobiome) contributes to immune system dysfunction (Figure 2) directly and indirectly by creating the conditions promoting EE/EED and by causing conditions (e.g., diarrheagenic infections especially) in the intestines which impede nutrient absorption. The latter conditions also contribute to reduction in oral vaccine responses (to be discussed below). Intestinal microbiome dysbiosis following infections such as SARS-CoV-2, norovirus, influenza, and HIV that are characterized by a shift toward an internal pathobiome, may significantly impair antiviral immune defenses. The dysbiosis compromises mucosal and systemic immunity, increasing susceptibility to secondary infections and reducing vaccine responsiveness.

Restoration of the intestinal microbiome appears to be critical for maintaining immune homeostasis, and interventions such as prebiotics, probiotics, and bioactive compounds (nutritionally driven microbiome modulation) have shown promise as adjunct strategies. These approaches may enhance the microbiome-gut-lung-axis, promote neuroimmune-endocrine crosstalk, improve oral vaccine immunogenicity, and support post-viral recovery outcomes (65). A role of the intestinal microbiome in modifying susceptibility and progression of neurodegenerative disorders also has been proposed (66, 67).

In normal (healthy) individuals, the mucosa of the intestine (comprised of epithelial cells, basement membrane, and lamina propria) provides a barrier between the intestinal lumen and the blood circulation which restricts the non-homeostatic passage (translocation) of pathogenic bacteria from the intestinal microbiome into the lymphatic system and systemic circulation (30, 68). As mentioned above, recurring enteric infections may result in EE/EED. A consequence of this syndrome is increased intestinal permeability that enables luminal pathogenic bacteria or bacterial byproducts to translocate into the lymphatic and systemic circulations (29). This may lead to subsequent immune activation and an intestinal and systemic inflammatory state, with multiple health effects, including inflammatory bowel disease and autoimmune disorders (30, 66, 69–71).

The dysbiosis of the intestinal microbiome leading to a shift to the pathobiome resulting in EE/EED (30) can lead directly to impacts on the immune system (27, 71). In fact, it appears that the critical interactions between the host immune system and the intestinal microbiota occur during the initial 3 years of life (68, 71), roughly the same as the critical time period for childhood linear growth and cognitive development (Figure 3). The finding that stunting prevalence is low soon after birth but increases with age, reaching a maximum prevalence by 20 months likely is a reflection of the fact that during the first 6 months of infancy, breastfeeding (especially the initial colostrum) and maternal IgG transferred transplacentally protect the infant from infection with enteric pathogens, support intestinal barrier maturation, and promote a beneficial early microbiome. In particular, secretory IgA (sIgA) and maternal IgG delivered through breast milk provide critical passive immunity in early life by coating mucosal surfaces, neutralizing enteric pathogens, and preventing their attachment and translocation across the infant gut barrier (72, 73). Together, these antibodies bridge the period before the neonate’s own immune system matures, reducing pathogen susceptibility and supporting healthy intestinal and immune system development. After weaning, however, infants lose this passive immunity while being exposed to contaminated and nutritionally inadequate food and at the same time behaviours such as hand-to-mouth activity, crawling, and contact with soil or animal feces result in an increase in exposure to pathogens through the fecal-oral route (15, 16, 39). The intestinal microbiome dysbiosis/pathobiome may result in long-lasting adverse impacts on the development and functioning of both the innate (i.e., monocytes, macrophages and innate lymphoid cells) and adaptive immune systems (i.e., B-cell and T-cell mediated immune responses), contributing to imbalanced immune homeostasis and susceptibility to infections and inflammatory diseases throughout the life of the individual (68, 71).

The various impacts of the synergistic malnutrition and infection cycle on the immune system have translated into poor responsiveness (take-off) to certain pediatric oral vaccines. The associations between enteric infections or EE/EED and diminished efficacy and duration of protection conferred by oral vaccines targeting diarrheagenic and non-diarrheagenic enteric pathogens have been reported repeatedly over the past two decades (4, 5, 50, 71–95, 132). The underperforming vaccines targeting diarrheagenic pathogens have included both killed and live oral cholera vaccines (71, 74), live oral rotavirus vaccines (39, 75, 77, 79, 81, 82, 84, 85, 90, 91, 93), live oral vaccines to enterotoxigenic *Escherichia coli* (92, 94), and live oral vaccines to *Shigella* (95, 132). An example of an underperforming vaccine targeting a non-diarrheagenic pathogen is the inactivated oral vaccine to poliovirus (39, 75–79, 81, 89).

**Figure 4:**
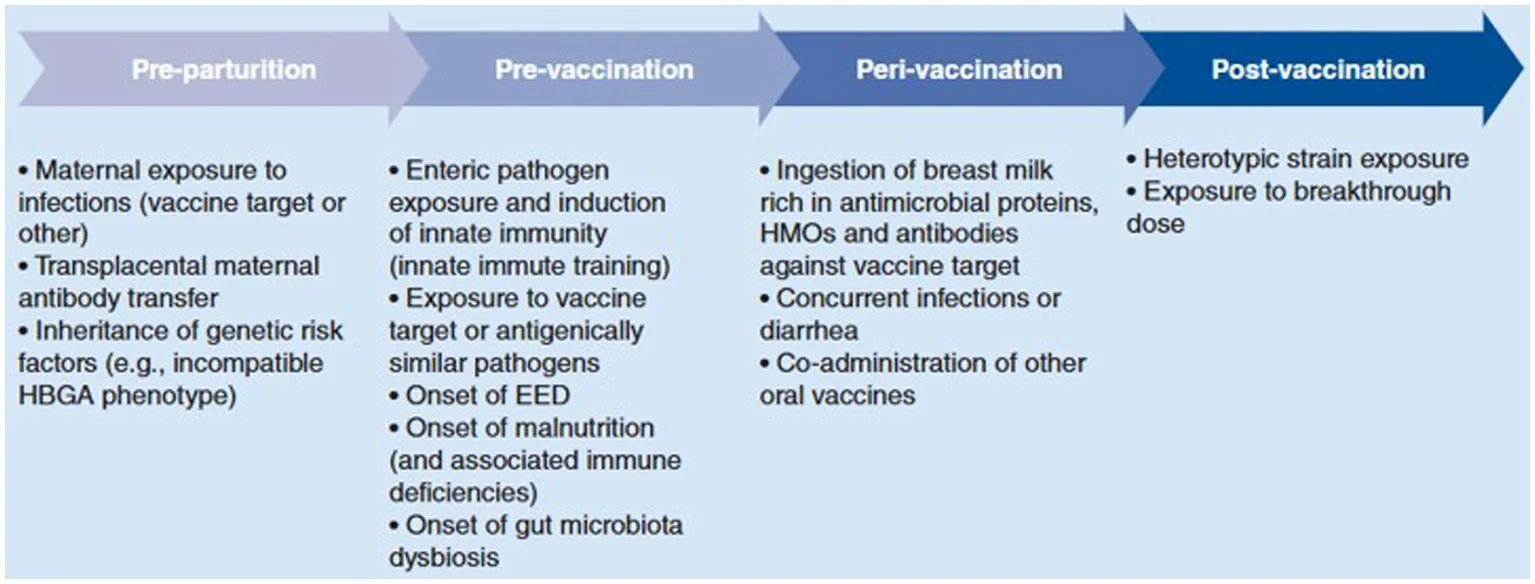
Factors contributing to diminished efficacy and duration of response to certain oral vaccines. From (96) per Creative Commons Attribution Non-commercial license 3.0. EED, environmental enteric dysfunction (environmental enteropathy); HBGA, histocompatibility blood group antigen; HMO, human milk oligosaccharide.

The diminished vaccine efficacy and duration of protection experienced by malnourished individuals in developing countries that has been reported for the diarrheagenic and non-diarrheagenic viruses mentioned above results from a number of factors (Figure 4), (96). The poor vaccine take-off for the enteric pathogens discussed above diminishes the value of the vaccine interventions for interrupting the synergistic malnutrition and infection cycle and leaves the impacted individuals relatively susceptible to the enteric pathogen assault to be described below.

### 3.5 Recurring enteric pathogen assault, a result of living in a pathobiome-dominated environment

As depicted in Figure 2, a recurring enteric pathogen assault appears to be an important contributor to the synergistic malnutrition and infection cycle. The enteric pathogens that appear to be most relevant to this cycle (10, 27) include non-enveloped and enveloped viruses, Gram-negative and Gram-positive bacteria, protozoans, and helminths (worms). A relatively complete list was assembled by Ijaz and Rubino (10) and has been reproduced in modified form here as Table 2. Note that while most of the listed pathogens are considered diarrheagenic, this is not true in all cases.

**Table 2:** Enteric pathogens considered relevant for the synergistic malnutrition and infection cycle [modified from reference (10)].

Pathogen type	Family/category	Example pathogen	Causative agent for	Diarrheagenic	Factors determining susceptibility to disinfecting agents
Virus	*Adenoviridae*	Enteric human adenovirus 41	Gastroenteritis	Yes	Non-enveloped; less susceptible
	*Astroviridae*	Human astrovirus	Gastroenteritis	Yes	Enveloped; highly susceptible
	*Caliciviridae*	Human norovirus	Gastroenteritis	Yes	Non-enveloped; less susceptible
	*Coronaviridae*	MERS-CoV, SARS-CoV, SARS-CoV-2	Gastroenteritis	Yes	Enveloped; highly susceptible
	*Parvoviridae*	Human bocavirus 1	Gastroenteritis	Yes	Non-enveloped; less susceptible
	*Picornaviridae*	Human enterovirus	Gastroenteritis	Yes	Non-enveloped; less susceptible
	*Reoviridae*	Rotaviruses	Gastroenteritis	Yes	Non-enveloped; less susceptible
	*Tobaniviridae*	Toroviruses	Gastroenteritis	Yes	Enveloped; highly susceptible
Bacteria	Gram negative	Enteropathogenic *Escherichia coli*	Infantile diarrhea	Yes	
		Enteroaggregative *E. coli*	Traveler’s diarrhea	Yes	
		*Salmonella typhi*	Typhoid fever	Yes	
		*Shigella dysenteriae*	Shigellosis (dysentery)	Yes	
		*Vibrio cholerae*	Cholera	Yes	
		*Yersinia enterocolitica*	Yersiniosis	Yes	
	Gram positive	*Campylobacter jejuni*	Campylobacteriosis	Yes	
		*Clostridium difficile*	Colon infection	Yes	Spores are less susceptible
Protozoa		*Cryptosporidium parvum*	Cryptosporidiosis	Yes	Oocysts are less susceptible
	Coccidian	*Cyclospora cayetanensis*	Cyclosporiosis	Yes	Oocysts are less susceptible
		*Entamoeba histolytica*	Intestinal amebiasis (amoebic dysentery)	Yes	
		*Giardia lamblia*	Giardiasis	Yes	Cysts are less susceptible
Helminths	Nematode	*Ancylostoma duodenale*	Old World hookworm	Yes	
	Nematode	*Ascaris lumbricoides*	Roundworm (ascariasis)	Yes	Ova are less susceptible
	Nematode	*Enterobius vermicularis*	Pinworm (enterobiasis)	No	
	Nematode	*Necator americanus*	Hookworm (necatoriasis)	No	
	Cestode	*Taenia saginata*	Tapeworm (taeniasis)	No	
	Nematode	*Trichuris trichuria*	Whipworm (trichuriasis)	Yes	

**Figure 5:**
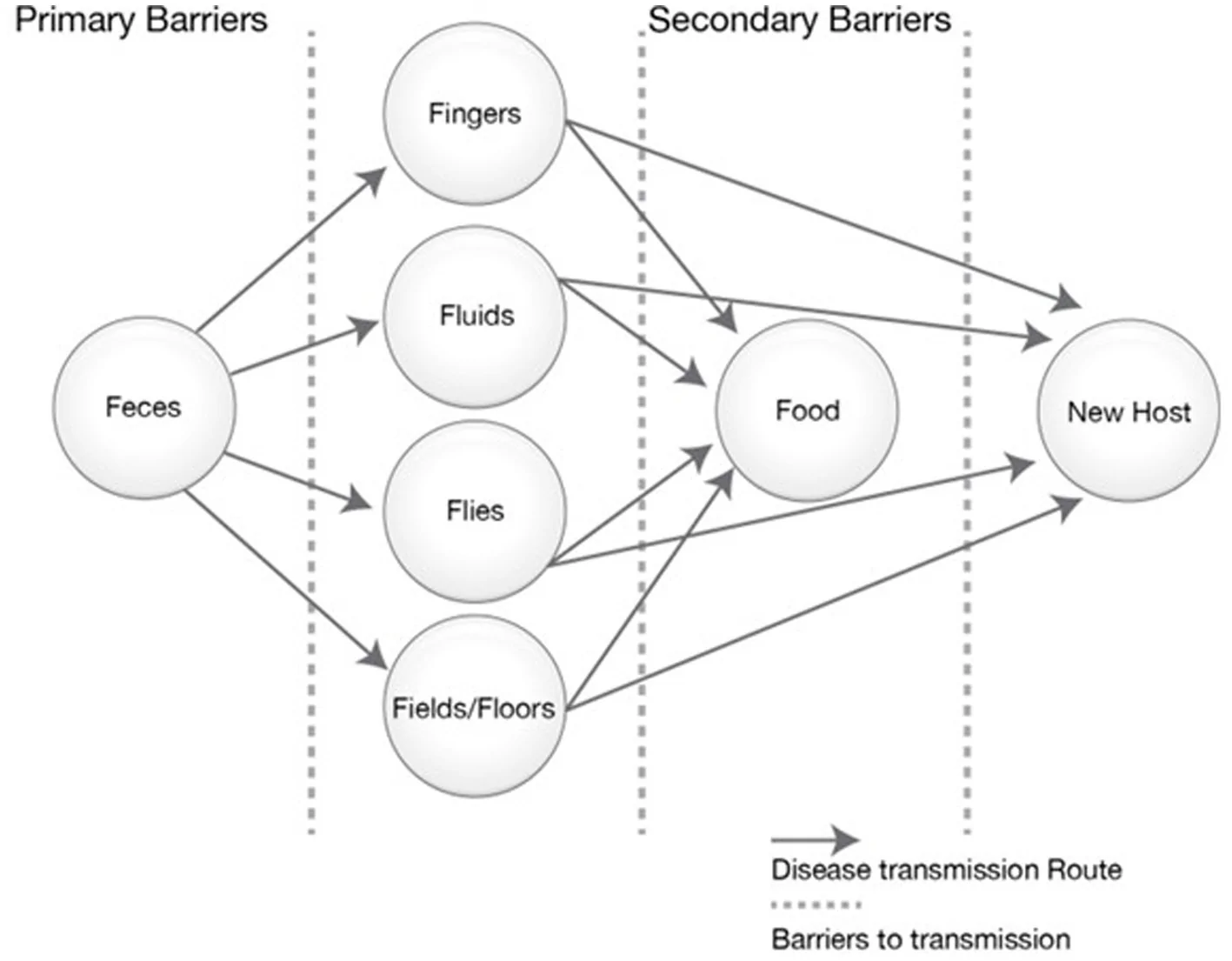
The F-diagram depicting various routes of transmission of infectious enteric pathogens to human hosts via the fecal-oral route. From (10) per Creative Commons Attribution Non-commercial license 3.0.

The recurring pathogen assault is especially impactful because of the increased susceptibility of malnourished infants to acquiring enteric infections, due in part to the immune dysfunction described above but also to the characteristics of the environment in which the infants live in resource-limited developing countries. The pathobiome-dominated environment is one in which pathogens may be present on a relatively continuous basis. These pathogens may be excreted by domestic livestock that are housed within the home, or by humans as a result of the open defecation that is practiced by almost two billion people globally, and predominantly in the Indian subcontinent (10). The acquisition of enteric infections through the fecal-oral route occurs as depicted in the so-called F-diagram, reproduced as Figure 5.

The associations between EE/EED and unsafe child feces disposal (96) or the mouthing by infants of pathogen-contaminated soil (geophagy) have been described previously (26, 96–98). Geophagy represents an important contributor to the fecal-oral route of infection transmission in resource-limited and developing countries (98). In addition, the inability to prevent exposure to the vectors (including house flies that are notorious for spreading enteric pathogens in developing nations) that transmit certain of the pathogens due to lack of screening, mosquito nets, etc., likely contributes to the acquisition of the infections caused by these pathogens (99, 100).

**Figure 6:**
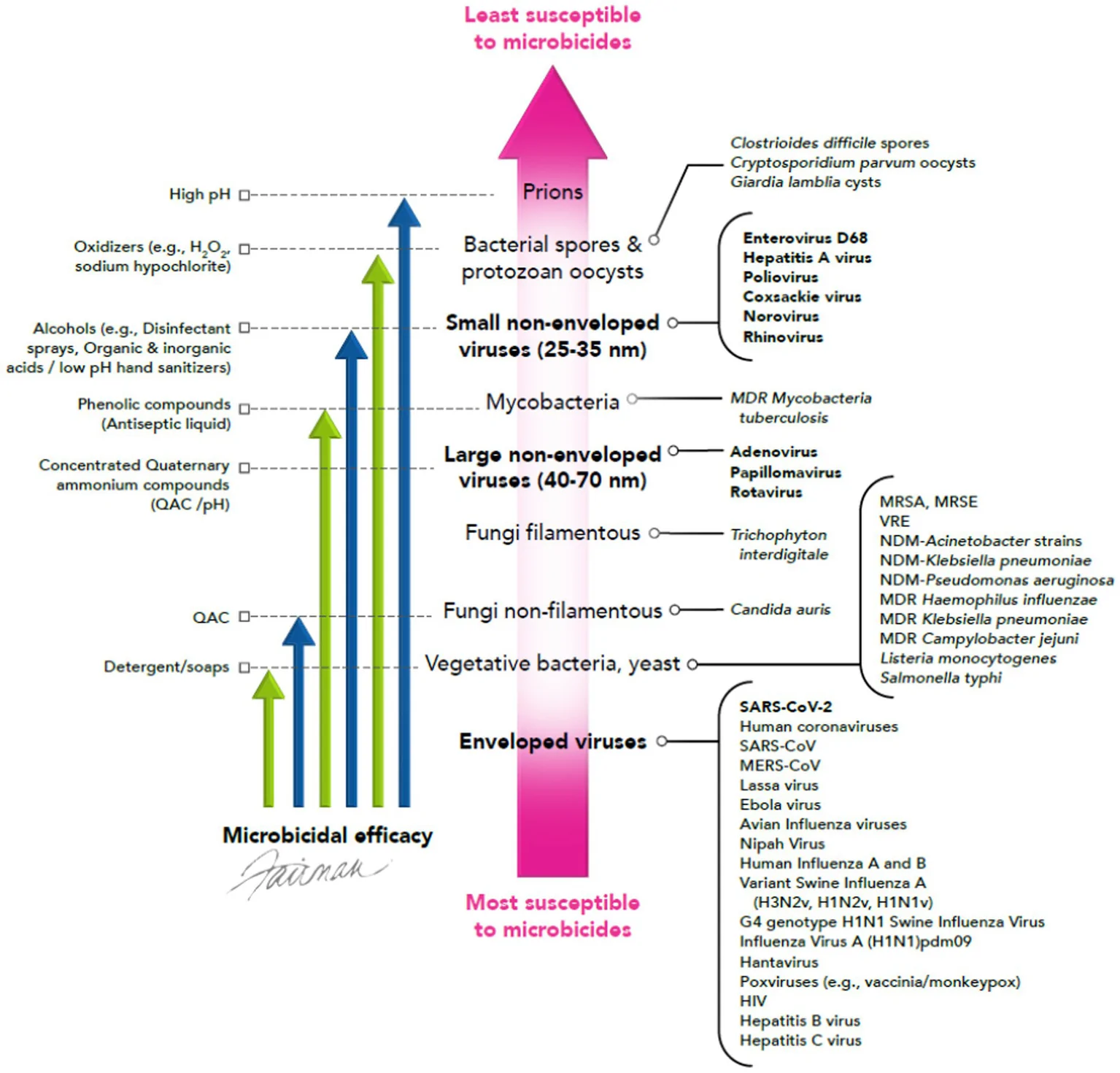
Hierarchy of sensitivity of pathogens to microbicidal actives [from reference (106)] © 2020, Fairman Studios, LLC. CC BY 4.0.

As indicated in Table 2, the various enteric pathogens differ with respect to their relative susceptibilities to microbicides (disinfectants, sanitizers, hygiene agents). This is a reflection of the so-called hierarchy of pathogen susceptibility to microbicidal actives (Figure 6) first proposed by Spaulding (101) and expanded upon by numerous investigators (102–106). In the absence of adequate disinfection, many of the enteric pathogens are capable of persisting in an infectious state in the environment over extended periods of time (Table 3), possibly contributing to transmission of infection to other family members or to re-infection of a given family member (especially relevant in the case of protozoan cysts/oocysts, helminth ova, and bacterial spores). Moreover, the continued presence of these various pathogens in infants intensifies the severity of enteric infections among malnourished children through mechanisms that disrupt immunity and alter gut microbiota (Table 4).

**Table 3:** Persistence of pathogens on inanimate surfaces in the absence of decontamination and time required to reach a noninfectious state [modified from reference (187)].

Pathogen (reference)	Infectivity	Fomite	*k*i (log_10_ reduction in titer/day)	Time needed for 3-log_10_ reduction^d^	Time needed to get below TCID_50_^e^
Non-enveloped viruses					
Adenovirus 40 (157)	150 TCID_50_	Non-porous^b^	0.264	11 d	14 d
Human astrovirus (158)	No data	Non-porous Porous^c^	1.1 2.4	2.7 d 1.3 d	No data No data
Feline calicivirus (159)	10–100 particles	Non-porous	0.286	10 d	21 d
Hepatitis A virus (157)	10–100 TCID_50_^a^	Non-porous	0.0667	45 d	90 d
		Porous	0.0667	45 d	90 d
Rotavirus p13 (157)	10–100 TCID_50_	Non-porous	0.0667	45 d	90 d
		Porous	0.0667	45 d	90 d
Enveloped viruses					
Coronavirus 229E (160, 161)	25–100 TCID_50_	Non-porous	0.50	6d	No data
		Porous	1.0	3 d	No data
Influenza virus (162)	2–790 TCID_50_	Non-porous	0.667	4.5 d	9 d
		Porous	1.00	3.0 d	6 d
Transmissible gastroenteritis virus (163)	No data	Non-porous	0.896	3.3 d	No data
Bacteria					
*Campylobacter jejuni* (164)	500 (165)	Non-porous	6.6	11 h	18 h
*Clostridium difficile* (166)	No data	Non-porous	0.0238	107 d	No data
*Escherichia coli* (167)	>10^5^ (166)	Non-porous	0.196	15 d	10 d
*Salmonella* spp. (168)		Non-porous	No published Ki		
		Porous			
*Shigella dysenteriae* (168, 169)	1–238	Non-porous	No published Ki		
		Porous			
Parasites					
*Ascaris lumbricoides* (187)	No data	Non-porous	0.0174	172 d	No data
		Porous	0.0160	188 d	
*Cryptosporidium parvum* (170)	10 (oocysts) (169)	Non-porous	0.0769	39 d	78 d
		Porous	0.0909	33 d	66 d
*Entamoeba histolytica* (187)	1,000 (171)	Non-porous	0.107	28 d	37 d
		Porous	0.143	21 d	28 d
*Enterobius vermicularis* (187)	No data	Non-porous	0.0938	32 d	No data
		Porous	0.115	26 d	
*Giardia muris* (170)	10 (cysts) (172)	Non-porous	0.100	30 d	100 d
		Porous	0.107	28 d	93 d

a d, days; h, hours; k_i_, inactivation rate constant; TCID_50_, tissue culture infective dose_50_.

b Non-porous surfaces include aluminum, stainless steel, and glass.

c Examples of porous surfaces include paper, latex glove, and cloth.

d The time in days for 1 log_10_ inactivation = 1/ k_i_. For the days required to obtain 3 log_10_ inactivation, multiply the days required for 1 log_10_ inactivation by 3.

e Assumes an initial burden of 10^6^ pathogens. The log_10_ reduction needed to achieve a bioburden 1 log_10_ lower than the lowest TCID_50_ is divided by k_i_ to arrive at the time required (days).

### 3.6 Environmental enteropathy, the result of recurring enteric pathogenic assault

**Figure 7:**
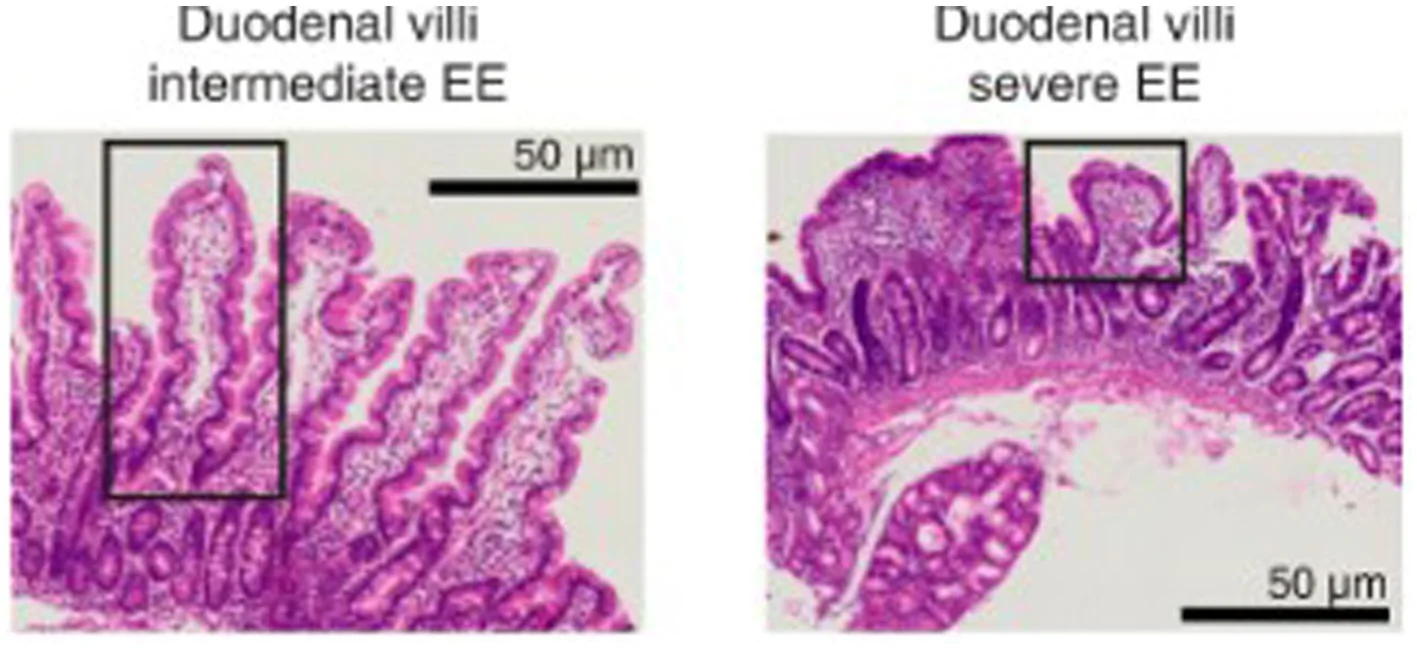
Microscopic images of hematoxylin and eosin-stained small intestine in patients with intermediate (left image, showing relatively normal appearance) and severe (right image showing villus blunting) environmental enteropathy (EE). Reproduced with permission from “scRNA-seq of the small intestine with and without EE. (A) H&E imaging of the small intestine with intermediate and severe EE. Intestinal villi with normal morphology (left image) and with severe blunting (right image) are highlighted in boxes” by Kummerlowe et al. (35).

As mentioned previously in this article, EE (also termed environmental enteric dysfunction, EED) is now recognized to be a sequella of recurring enteric pathogen exposure and is a subclinical syndrome considered to be more important in the context of childhood stunting than diarrhea itself (25–37) (Figure 2). The clinical syndrome involves structural manifestations which directly contribute to functional deficiencies in nutrient absorption from the small intestine. These structural changes include the blunting (decreased height) of small intestinal villi (Figure 7) and increased depth of small intestinal crypts (crypt hyperplasia), as well as indications of inflammation (lymphocytic infiltration of the lamina propria and increases in intraepithelial lymphocytes) (25, 30, 33–35, 37).

**Figure 8:**
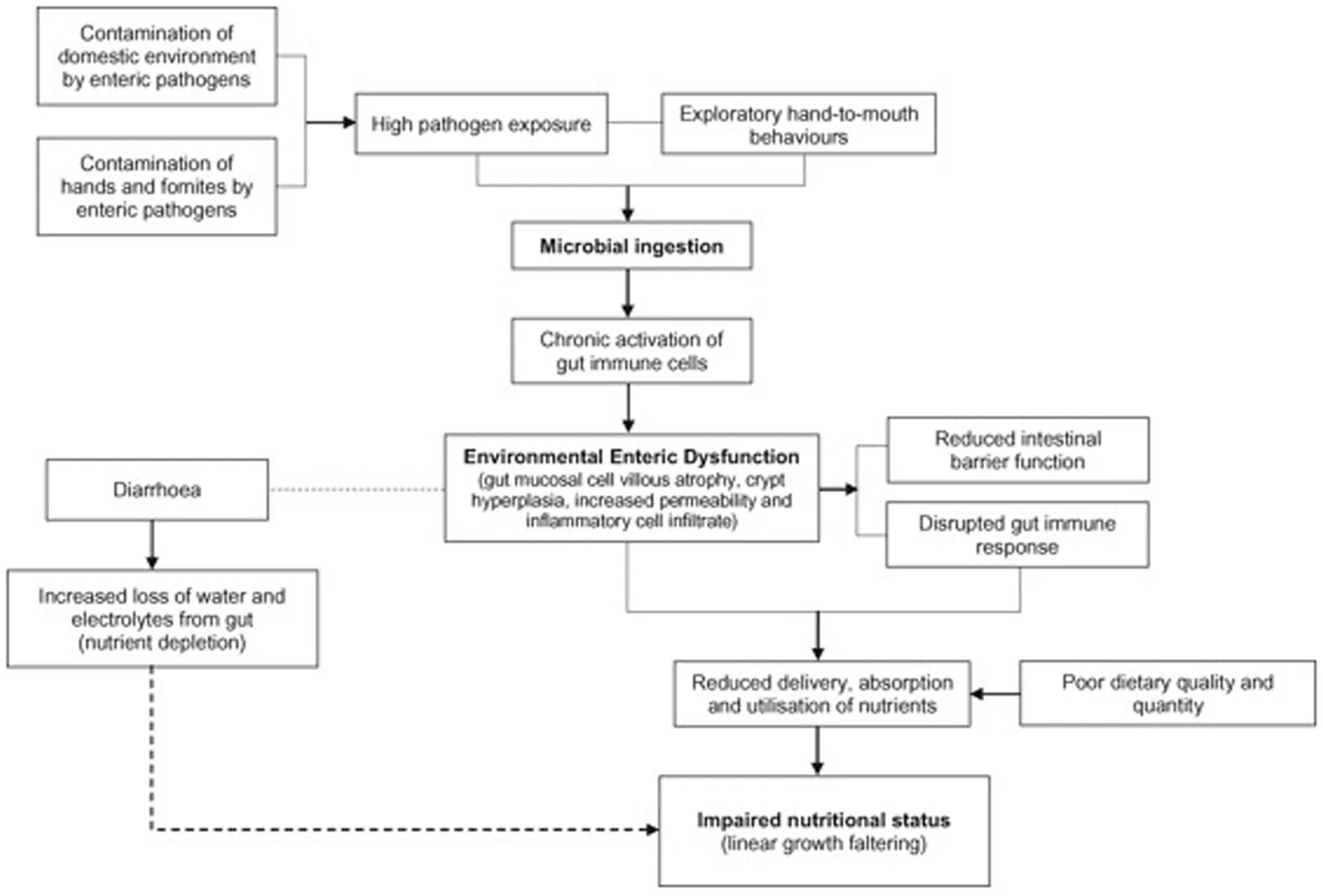
A non-cyclic depiction of the linkage between environmental enteric dysfunction and growth stunting (from reference 33). The figure is created from an open access souce Creative Commons Attribution-NonCommercial-NoDerivs licence (http://creativecommons.org/licenses/by-nc-nd/4.0/).

The associated functional changes include adverse impacts on the absorptive function of the small intestine, increased intestinal permeability, and a chronic hyperimmune state characterized by inappropriate engagement of the innate and adaptive immune responses of the intestines (25, 30, 33–35, 37, 107). The link between EE/EED and linear growth stunting is portrayed schematically in Figure 8 [reproduced from reference (33)]. Although this is a non-cyclic portrayal, most of the elements of the synergistic malnutrition and infection cycle discussed in the current article may be found within the figure, further highlighting the difficulty in determining the start point of this vicious cycle.

While endoscopy and intestinal biopsy remain the optimal diagnostic indicators of EE, other less invasive biomarkers also may be monitored (19, 30, 36, 39, 40, 107). A commonly used biomarker that is based on changes in intestinal permeability is the ratio of lactulose to mannitol in the urine, with higher ratios being indicative of increased permeability and malabsorption (15, 19, 25, 29, 36, 107, 108). Environmental enteropathy is considered to be reversible (9, 25, 75, 107), provided that the pathogen assaults and other conditions predisposing to enteropathy are prevented from recurring. In addition, certain therapeutic interventions have been proposed for addressing the structural and functional changes observed in EE/EED (discussed in the following section).

### 3.7 Interrupting the synergistic malnutrition and infection cycle

**Figure 9:**
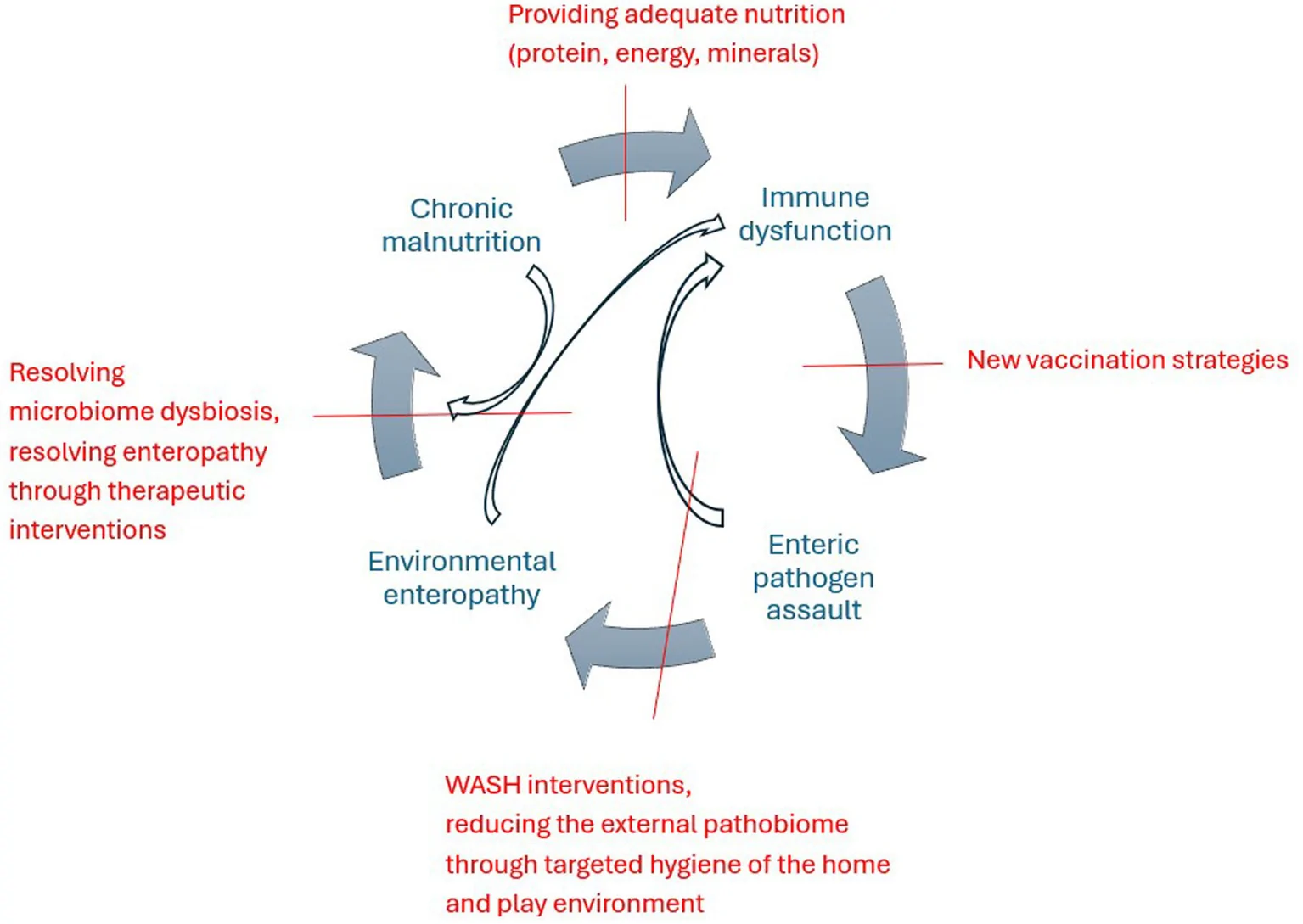
Multiple pharmaceutical and non-pharmaceutical interventions (red font) may be required to resolve environmental enteropathy and to interrupt the synergistic malnutrition and infection cycle and associated childhood stunting.

How are we to interrupt the synergistic malnutrition and infection cycle and reduce the associated stunting syndrome? Historically, the interventions proposed and evaluated (Figure 9) have included the following: (1) nutritional/caloric interventions; (2) interventions targeting water quality and household/community sanitation practices (WASH); (3) interventions specifically aimed at reducing the exposure of infants to the pathobiome-dominated environment (“baby WASH”); and (4) pharmaceutical (therapeutic) interventions (vaccination, and therapeutics targeting the dysbiosis and enteropathy). The successes or failures of these interventions typically have been monitored through infant anthropometry (weight-for-height, weight-for-age, height-for-age, etc.), biomarkers of EE/EED, and enteric pathogen loads on hands and on environmental surfaces, or in actual patient diagnostic samples. The potential impacts of hygiene interventions on pathogen exposure risk may be estimated through application of Quantitative Microbial Risk Assessments (QMRA). It has been proposed that EEG rhythmic activity might be used as an indicator of cognitive decline due to early childhood malnutrition (109).

#### 3.7.1 Nutritional interventions

A logical starting point for addressing malnutrition-associated stunting seem to be improving the quality and quantity of the nutritional resources (including both protein/energy foods as well as minerals/vitamins/metals) available to infants, especially during the critical time from birth through age 2, and to mothers during the gestational period. Theoretically, nutritional interventions should provide the energy required to mount immune responses to invading pathogens, and the nutrients needed to support linear growth and cognitive development. Unfortunately, it appears that nutritional interventions alone have not been successful in reducing the stunting syndrome (17, 110–114). The analogy of the futility of adding water into a bucket with a hole in it (i.e., the *leaky bucket*) (4) is likely applicable to this situation. Improving nutrition without addressing the other contributors to the synergistic malnutrition and infection cycle (Figure 9) is not sufficient to reduce stunting. Due to the nature of the synergistic malnutrition and infection cycle (Figure 9), attempts to remediate single outcomes (contributors of the cycle), such as the nutritional deficits, clearly are unable to interrupt the entire cycle. The interdependencies between the various contributors (components) must also be addressed (108) in a holistic manner.

#### 3.7.2 WASH interventions

The realization that nutritional supplementation alone was not leading to the expected reductions in childhood stunting led to efforts focusing on remediating poor water quality and quantity, and home/community sanitation interventions involving implementing technologies and behaviours intended to safely contain excreta and preventing human contact with excreta. The latter has included implementation of hand washing with soap and water after defecation and before handling and preparing food or eating (16, 18). These “WASH” interventions have been designed to interrupt the fecal-oral transmission of many of the enteric pathogens (Figure 5). The expectation was that these interventions, implemented alone or in combination with adequate nutrition, might be sufficient to reduce growth stunting associated with malnutrition.

A variety of recent trials have suggested that these WASH interventions have not meaningfully reduced the occurrence of the enteric infections associated with the stunting syndrome or the stunting itself (6, 14, 16, 19–23, 30). Inconsistent reporting of WASH implementation details has been cited as one barrier to the evaluation and comparison of WASH field trial results (188). The WASH Benefits Bangladesh trial (189), for example, tested whether household interventions could improve child growth by interrupting fecal-oral transmission. Although nutritional supplementation modestly improved growth and survival, none of the WASH interventions (alone or in combination) produced measurable effects on linear growth, despite reductions in reported diarrhea. The lack of synergy with nutrition suggests that diarrhea reduction alone is not enough to disrupt EE/EED or its developmental consequences, thus highlighting the limited effectiveness of household-level WASH interventions in high exposure risk settings. A further explanation lies in zoonotic exposures, particularly in poor WASH settings such as rural areas. Evidence from rural Bangladesh (190), shows that animal feces, particularly from chickens, represent a significant source of environmental contamination and thus a major contributor to EE/EED. Without tackling these exposures, by preventing animal waste from contaminating domestic environments and from being within infants’ reach, WASH interventions cannot sufficiently reduce pathogen burden to break the synergistic cycle of infection, immune activation, and impaired growth. This suggests that broader integrated strategies targeting multiple contributors to the synergistic malnutrition and infection cycle (Figure 9) also must be considered. These may include targeted hygiene in the home, play areas, and school settings of impacted children.

#### 3.7.3 Baby WASH interventions (environmental hygiene)

Budge et al. (33) summed up the current thinking on childhood stunting very succinctly: “Although it might seem obvious that the risk of pathogen exposure should be a major consideration in intervention design, so far WASH programs and ECD [early childhood development] interventions, such as the Essential Package from Save the Children and the Care for Child Development Package from UNICEF, have not specifically tackled the pathogen burden encountered by babies and infants in their home and play environments.” We agree with this line of thought and the need for interventions specifically targeting this aspect of the synergistic malnutrition and infection cycle (15, 16, 19, 26, 28, 33, 115).

The importance of interrupting this recurring pathogen assault has been recognized by several researchers. As early as 2013, Ngure et al. (115) observed that WASH interventions did not effectively eliminate fecal-oral transmission of bacteria among infants and young children, stating that “New interventions and programs are needed to address these environmental health risks that potentially diminish the benefits achievable for childhood health and growth from improved dietary interventions.” In 2014, Prendergast and Humphrey (15) suggested that “Interventions need to be targeted at the pathways through which faeco-oral transmission [of enteric pathogens] commonly occurs in infancy, such as exploratory behaviour leading to ingestion of animal faeces and soil, known to be highly contaminated in settings where people live with livestock in the so-called infant-targeted approach termed “baby WASH.” Other terms that have been used for this approach include “environmental hygiene” (19) and “environmental sanitation” (116), which seem appropriate since it is the immediate environment of the infant (living and play spaces) that is the proposed target for hygienic interventions. Cumming and Cairncross (16) observed in their 2016 article that interventions targeting critical exposure points for young children should be prioritized along with improvements in nutrition. Such exposure points include improving infant food hygiene and controlling or supervising exploratory play to limit exposure to contaminated soil, fomites and play objects as well as ensuring that child feces are disposed of safely (15, 16, 116, 117).

In their 2016 article, Mbuya and Humphrey (28) suggested that “WASH interventions seeking to prevent EED [environmental enteric dysfunction] should address specific pathways through which fecal-oral transmission occurs in the first 2 years of a child’s life.” These authors also provide a list of proposed interventions based on the baby WASH approach that might be explored, among which was providing a clean play and infant feeding environment (28). These interventions take into account the fact that there are distinct transmission routes that specifically pertain to infants that must be considered, such as pathogen contamination from domestic animals sharing household spaces in which young infants play and sleep, a factor considered to be more relevant during the first thousand days of a child’s life than contaminated drinking water (33).

The baby WASH/environmental hygiene interventions thus are designed to reduce the behaviours associated with geophagy and to reduce the pathobiome of the immediate living environments of the infants. Recognizing that diarrheagenic *E. coli* has been recovered on surfaces and objects such as toys and balls that an infant regularly encounters as part of play (96), and the fact, discussed earlier in this article (Table 3), that a number of the relevant enteric pathogens may survive for extended periods of time (weeks to months) on fomites or in soil, these exposure routes represent critical, undisrupted contributors to the synergistic malnutrition and infection cycle and an important gap for appropriate interventions (33). Certain life stages of parasites and helminths, especially, are capable of remaining infectious for extended periods of time in feces and in soil contaminated with feces (118). Additional evidence is available to support the baby WASH (environmental hygiene) approach. For instance, Lin et al. (119) observed in 2013 that children living in clean household environments had lower frequencies of parasitic infections, improved gut function, and improved growth, compared with similar children living in contaminated environments. The term “contaminated environments” was, in this case, defined as a household with median *E. coli* levels in water of ≥10 CFU/100 mL, inadequate sanitation, and nonhygienic handwashing conditions (119). In 2017, Morita et al. (98) suggested that childhood mouthing of objects and soil (geophagy) represents an important pathway for exposure to enteric pathogens and for acquiring infections promoting environmental enteropathy. George et al. (120) found in 2020 a high fecal contamination of play spaces and food and on surfaces and objects mouthed by children in a study conducted in rural areas of The Democratic Republic of the Congo.

**Table 4:** Persistence *in vivo*, impact, and mechanistic outcome of enteric pathogen infection in malnourished children.

Pathogen (reference)	Persistence in vivo	Physiological impact	Mechanistic outcome
Bacteria			
*Salmonella* (173, 174)	High (systemic spread)	Systemic infection, growth impairment	Dysbiosis, immune evasion linked to *Salmonella* type III secretion system (T3SS)
Enteroaggregative *Escherichia coli* (175)	Chronic (biofilm formation)	Impaired growth, gut barrier dysfunction	Toxin production, microbiota disruption, dysbiosis, immune evasion
*Klebsiella pneumoniae* (176, 177)	Recurrent (inflammatory)	Bloody diarrhea, stunting	Colonic invasion via type VI secretion system (T6SS) gene expression, and immune evasion
Viruses			
*Rotavirus* (178)	Moderate to high (increased shedding)	Diarrhea, reduced vaccine efficacy	Altered microbiota, poor vaccine response
*Norovirus* (179–181)	High (enhanced evolution)	Severe diarrhea, immune suppression	Impaired immunity, dysbiosis, higher transmission, nosocomial infections
*Adenovirus* (182, 183)	Prolonged shedding	Diarrhea, dehydration	Delayed clearance, epithelial tropism
*Astrovirus* (184)	Extended duration	Mild to moderate diarrhea	Low immunogenicity, mucosal suppression
Parasites			
*Cryptosporidium* (185)	Chronic (intracellular)	Watery diarrhea, malabsorption	Intracellular parasitism, *Cryptosporidium parvum,* viral protein-directed immune resistance /evasion
*Giardia lamblia* (186)	Recurrent or asymptomatic	Growth faltering, malabsorption	Antigenic variation, immune modulation

Accordingly, the baby WASH approach emphasizes the need for creation of a hygienically clean environment in which babies and infants can play that reduces the potential fecal-oral transmission routes for recurring enteric pathogens (33). An example of trialing out such an intervention was conducted by Budge et al. (121), considered to be the first randomized controlled feasibility trial of a baby WASH household play space in rural Ethiopia, which aimed to reduce infants’ exposure to contaminated soils and animal feces. The intervention was associated with a marked reduction in reported diarrhea prevalence among infants but did not reduce *Campylobacter* infection, which potentially suggests persistent exposure via other routes. Ensuring hygienically safe baby WASH play spaces could therefore contribute to disrupting the multiple overlapping pathways driving enteric infection and EE/EED, thus highlighting the need for integrated, community-level strategies.

While baby WASH interventions such as protective play spaces (121) aim to reduce direct contact with contaminated environments, evidence also shows that educational approaches are critical in shifting behaviours that drive exposure risks. Negussie et al. (122) demonstrated that multimedia education through radio, community campaigns and culturally resonant campaigns through drama and music substantially improved reported practices, including safe child feces disposal, hand washing and food hygiene. This highlights the potential of tailored behaviour change strategies to shift entrenched norms around child hygiene. Integrating educational approaches with structural baby WASH improvements offers greater potential to embed and sustain the behavioural changes needed to sufficiently disrupt EE/EED.

Creating hygienically safe play, living, and learning spaces for infants may require the targeted application of microbicides with efficacy towards each of the enteric pathogens known to be associated with EE/EED (see Tables 2, 3). As indicated in Figure 6, certain of these enteric pathogens are known to exhibit relatively low susceptibility to microbicidal active ingredients, so selection of stringent broad-spectrum microbicides will be required.

The impact of such targeted environmental hygiene interventions can be challenging to assess. While epidemiological studies can be used to assess the impact of interventions on observed infections or changes in antibody titers to specific pathogens, these require significant resources and the interventions to be evaluated can be difficult to formulate or select in advance.

#### 3.7.4 Application of quantitative microbial risk assessments (QMRA) for assessing potential impact of targeted interventions

An approach that circumvents the issues described above is the application of QMRA. This approach uses a combination of microbicide dose-inactivation response data and pathogen exposure data to estimate the probability of acquiring an infection with that pathogen. The QMRA approach has been used more recently, and guidance has been developed by the World Health Organization for application of QMRA to the setting of drinking water treatments and food safety (123, 124). This approach has also been used to assess risk from exposure to pathogen-contaminated environmental surfaces (fomites) (125, 126) and risk of pediatric infections attributable to soil ingestion in Mozambique (127). Provided that data on exposure levels for a given pathogen for a specific route (e.g., ingestion of soil, water, or food) are available, the QMRA approach enables the quantitative estimation of probabilities of acquiring an infection with or without a proposed targeted hygiene intervention. In this manner, the interventions which should have the greatest impact on infection reduction can be determined. The approach can also be used to set targets for the required reduction of pathogen concentrations on a surface to achieve a desired reduction in the associated infections (128).

#### 3.7.5 Pharmaceutical (therapeutic) interventions

A number of pharmaceutical interventions have been considered for interrupting the malnutrition and infection cycle and reducing EE/EED and, ultimately, the stunting syndrome. Reducing enteric infections resulting from the recurring pathogen assault through vaccination has been attempted. Most important among these may be the rotavirus and cholera vaccination programs (71). Vaccines are still under development for *Shigella* (8, 71, 92, 132), enteropathogenic *E. coli* (8, 71, 92, 94), *Campylobacter jejuni* (191), *Clostridium difficile* (129), *Entamoeba histolytica* (130), and *Cryptosporidium* spp. (131). As mentioned previously in this article, the efficacies of the existing vaccines as well as of the developmental candidates for *Shigella* and enteropathogenic *E. coli* have, unfortunately, been suboptimal (exhibiting poor take-off and reduced duration of protection) in children suffering from malnutrition and EE (4, 5, 71–92, 132). Approaches that might be taken to improve the efficacies of oral vaccines (use of novel adjuvants or coadministration with mucosal repair nutrients, etc.) need to be developed and evaluated (11, 133, 134).

Aside from vaccination against enteric pathogens, other therapeutic interventions have been or are being considered (8, 11, 12, 25, 27, 30, 31, 68, 135–143). These include the amino acids glutamine or alanylglutamine and arginine or citrulline (8, 25, 27, 31, 68), the immunomodulatory or immunosuppressive agents mesalazine (136) and budesonide (137, 138) and teduglutide, a glucagon-like peptide-2 analogue (136, 137) for targeting the inflammatory disruption and structural changes in EE/EED. Additional therapeutic strategies include zinc supplementation (8, 11) for reducing the impact of diarrhea; targeted antimicrobial therapies for specific pathogens (11, 12, 25, 27, 30, 31); and probiotics for remediating the dysbiosis (pathobiome) associated with EE/EED (11, 12, 27, 31, 49, 138–143). Of these therapeutic interventions only the latter intervention (remediating the dysbiosis) appears to have shown effectiveness in reducing the incidence of childhood stunting (135). This may reflect, in part, the complex interplay between the intestinal microbiome, host physiology, and viral infections (139, 140). Supporting this is a proposal to apply nutrigenomics to determine critical genes associated with metabolic pathways that may require micronutrients as cofactors (143). It has been suggested on the basis of animal studies that the nutritional dysbiosis mentioned above must be corrected during the prenatal period as well as during the postnatal period for effective prevention of stunting to be possible (144).

#### 3.7.6 Epigenetic reprogramming through nutritional interventions and supplementation

Emerging clinical and experimental evidence suggests that nutritional interventions may partially reverse or mitigate epigenetic alterations resulting from malnutrition, thereby influencing long-term health outcomes. The degree of this reversibility, however, is contingent upon several factors, including the developmental timing of intervention, the specific nutrients involved, and individual pathobiological and environmental contexts.

For instance, a robust body of research demonstrates that supplementation with methyl-donating nutrients such as folate, choline, betaine, methionine, and B vitamins during critical developmental periods can restore aberrant DNA methylation patterns and improve gene expression profiles associated with metabolic and neuropsychiatric regulation (58, 145–147). Interventions administered during gestation or early postnatal life appear to be particularly effective, as these developmental periods are sensitive windows during which nutritional modulation can recalibrate epigenetic programming and reduce the risk of non-communicable diseases in later life (142, 146).

Folic acid, the synthetic form of folate, is found naturally in foods such as leafy green vegetables. It is a strongly recommended supplement for women who are pregnant or planning pregnancy, as adequate folic acid intake has been shown to significantly reduce the incidence of neural tube defects. Following the adoption of folic acid supplementation in the United States, rates of congenital neural tube anomalies have markedly declined. Guerrant *et al.* (148) demonstrated using murine models that even mild zinc deficiency enhances the virulence of enteric pathogens, including *Campylobacter*, ETEC, *Shigella*, EAEC, and EPEC. Globally, it is estimated that 17% of the human population is zinc deficient, a condition strongly associated with growth stunting. Oral zinc supplementation has proven effective in restoring zinc levels among deficient individuals, and the incidence of diarrhea and its severity was found to be reduced in children receiving zinc supplementation daily for 4 months (149).

Moreover, dietary patterns emphasizing nutrient density and bioactive compounds (such as the Mediterranean diet) are linked with slower epigenetic aging and diminished susceptibility to chronic disease (149). Such diets may attenuate adverse epigenetic processes, including inflammation and oxidative stress, while promoting restoration of metabolic homeostasis even in adulthood (150, 151).

Nevertheless, the reversibility of epigenetic modifications is inherently constrained. Certain changes, particularly those established during early childhood development, may persist over the course of a lifetime and contribute to heightened disease vulnerability despite subsequent dietary improvement (63, 145, 151). The efficacy of nutritional interventions is further influenced by interindividual variability, including genetic predisposition, gut microbiome composition, and cumulative environmental exposures (152, 153).

Although childhood malnutrition is multifactorial, this section highlights a study examining the relationship between EE/EED and malnutrition as a potential avenue for therapeutic intervention. The timing of these exposures is critical. As reflected in Figure 3, the period from gestation through the first 2 years of life represents a particularly vulnerable window for growth, immune system development, and intestinal health. During this period, malnutrition, recurrent enteric infection, and immune dysfunction can become mutually reinforcing. It is therefore difficult to separate the effects of inadequate nutrition from those of repeated intestinal infection and EE/EED, because these processes interact in a cycle: poor nutrition weakens immune defenses and intestinal integrity, while recurrent infection and intestinal inflammation impair nutrient absorption and growth.

The Study of Environmental Enteropathy and Malnutrition, or SEEM, helps clarify this relationship (53, 54). SEEM followed children from birth to 24 months in rural Pakistan to identify factors associated with growth outcomes to better understand predictors that could inform management strategies for environmental enteropathy. The primary outcome was length/height-for-age Z score, or HAZ, at 24 months of age, used as a measure of linear growth and stunting. Secondary outcomes included weight-for-length/height Z score, or WHZ, as a measure of wasting, and weight-for-age Z score, or WAZ, as a measure of underweight. The study also examined genes and biological pathways associated with the pathogenesis of EE/EED (53, 54).

Within the SEEM-AKU birth cohort, researchers followed both malnourished children and controls with adequate growth through 24 months of age. Their findings provide additional evidence that EE/EED is not simply a nutritional deficiency state, but a broader biological process involving inflammation, intestinal injury, altered growth signaling, and changes in epithelial function. The analysis of genome-wide DNA methylation, or DNAm, in duodenal biopsy specimens from children with EE/EED and controls identified thousands of differentially methylated regions between the two groups, including hypermethylation in genes involved in transcriptional regulation, wound healing, epithelial adhesion, chromatin modification, differentiation, and cellular proliferation (53, 54).

Taken together, these findings deepen the understanding of the synergistic malnutrition and infection cycle, and suggest that early-life environmental exposures may alter intestinal biology at the epigenetic level, potentially affecting how genes involved in repair, growth, immune response, and epithelial integrity are expressed. This is significant because development, aging, diet, and gut microbes are each known to influence intestinal DNAm. Although improving nutrition remains essential, SEEM findings emphasize that nutritional intervention alone may not fully reverse the biological consequences of EE/EED once intestinal and epigenetic changes have occurred.

Accordingly, the SEEM study (53, 54) points toward an important future direction: targeting intestinal DNAm and related pathways as potential therapeutic strategies for EE/EED. Because the consequences of EE/EED may persist into childhood and adulthood despite nutritional, pharmacologic, water, sanitation, and hygiene interventions, DNAm may represent a promising area for further research. More broadly, SEEM reinforces that childhood stunting and malnutrition should be understood not as isolated nutritional outcomes, but as the result of overlapping social, infectious, immunologic, microbial, and epigenetic processes that begin early in life and may have long-term consequences.

To summarize, evidence supports that targeted nutritional strategies, particularly those enhancing methyl donor availability and overall diet quality, may beneficially modulate epigenetic landscapes and improve health trajectories. Early-life interventions remain most effective, as many developmental epigenetic alterations are only partially reversible once established.

Aside from the intervention categories discussed above, a variety of suggestions from researchers for potential new approaches have been offered. An example is the suggestion by Sinharoy et al. (154) of a potential link between air pollution and stunting. Early detection approaches for stunting have also been proposed (155). It is too early to say much about the utility of these proposals. The holistic approach to implementation of interventions advocated here needs to be coupled with education targeting the behavioural changes needed to sustain the goals discussed above. To achieve this, there needs to be integration and cooperation of policymakers/governments for supporting and sustaining these interventions over the long term. This, in turn, will contribute not only to childrens’ long-term physical and neurocognitive development but also to bringing the economic prosperity of the target populations to levels approximating those of developed nations (156).

## 4 Limitations and future needs

The review underlying this Hypothesis and Theory article was not intended to represent a thorough review of the literature pertaining to causation of stunting by malnutrition but was intended to address the various components of the synergistic malnutrition and infection cycle with enough detail to inform the reader of the complexities of the cycle and the suspected interdependencies between components of the cycle. Here, we have focused on the interplay between malnutrition, the gut microbiome, various pharmaceutical and non-pharmaceutical interventions, and epigenetic changes that may contribute to stunting. This article necessarily represents the authors’ viewpoints, and evidence supporting these viewpoints may not at present exist. Appropriate field studies designed to test the hypothesis advocated in this article, that disrupting the ongoing pathogen assault is required for reducing the stunting associated with the synergistic malnutrition and infection cycle, must be conducted. Moving forward, research and policy efforts should prioritize coordinated interventions at the individual, community, national, and international levels—aimed not only at preventing malnutrition but also at mitigating its profound health and developmental consequences worldwide.

### 4.1 Thoughts about possible designs for field trials to support the baby WASH hypothesis

Future studies should evaluate whether reductions in infant EE/EED and asymptomatic enteric pathogen burden mediate improvements in EE/EED biomarkers and infant development. The baby WASH hypothesis proposes that interventions targeting infant-specific pathogen exposure pathways within household and play environments might be more effective in reducing EE/EED and its associated consequences than conventional WASH interventions alone. Cluster-randomised controlled trials (RCT) and longitudinal birth cohort studies could provide rigorous evaluation frameworks to test this hypothesis in the future. The former could compare conventional WASH interventions (water quality improvements, sanitation and handwashing promotion) with baby WASH interventions specifically designed to reduce infant exposure to fecal contamination through contaminated soil, fomites, toys, food, animal feces and household surfaces. An additional intervention arm combining baby WASH, conventional WASH and nutritional support could evaluate whether integrated approaches provide greater benefits than single interventions.

Longitudinal studies may be conducted to follow children from birth to 2 years of age in order to provide complementary evidence regarding the temporal relationships between environmental pathogen exposure, EE/EED development, and growth outcomes. Using a combination of environmental, biological, and clinical indicators to assess baby WASH interventions could amplify the effectiveness of this intervention. Environmental measures may include quantification of enteric pathogen contamination on household surfaces, infant toys, caregiver hands, food preparation areas, and play environments. Molecular detection methods, such as PCR, may also be used to measure enteric pathogen burden, including diarrheagenic *Escherichia coli*, *Shigella* spp., *Campylobacter* spp., *Cryptosporidium* spp., *Giardia* spp., rotavirus, and norovirus. Biological indicators may include biomarkers of EE/EED, particularly the urinary lactulose ratio, which reflects intestinal permeability and malabsorption. Additional outcomes could include diarrhea incidence, vaccine responsiveness, and anthropometric measures such as height-for-age-Z-scores and weight-for-age-Z-scores. Together, these indicators could inform whether reductions in environmental pathogen exposure are associated with improvements in intestinal health and infant linear growth. Employing RCTs and longitudinal studies could provide further evidence regarding whether baby WASH interventions can more effectively interrupt the cycle of enteric pathogen exposure, EE/EED and childhood stunting than traditional WASH interventions alone.

### 4.2 Public health and policy implications for LMICs

Implementing an integrated maternal and child health and environmental hygiene package should reduce infection burden, restore immune homeostasis, improve vaccine performance, and accelerate gains in linear growth and neurocognition. For policymakers, these investments are high value: they lower downstream healthcare costs, enhance school readiness, reduce absences and improve attainment, and expand the future workforce’s productivity, directly contributing to economic growth. Success requires coordinated action across health, water/sanitation, education, and agriculture sectors; inclusion in primary-care platforms (antenatal care, immunization programs, growth monitoring); and financing that enables scale-up, quality assurance, and monitoring (e.g., standardized hygiene indicators, EE/EED biomarkers, and child development metrics).

### 4.3 Call to action

**Figure 10:**
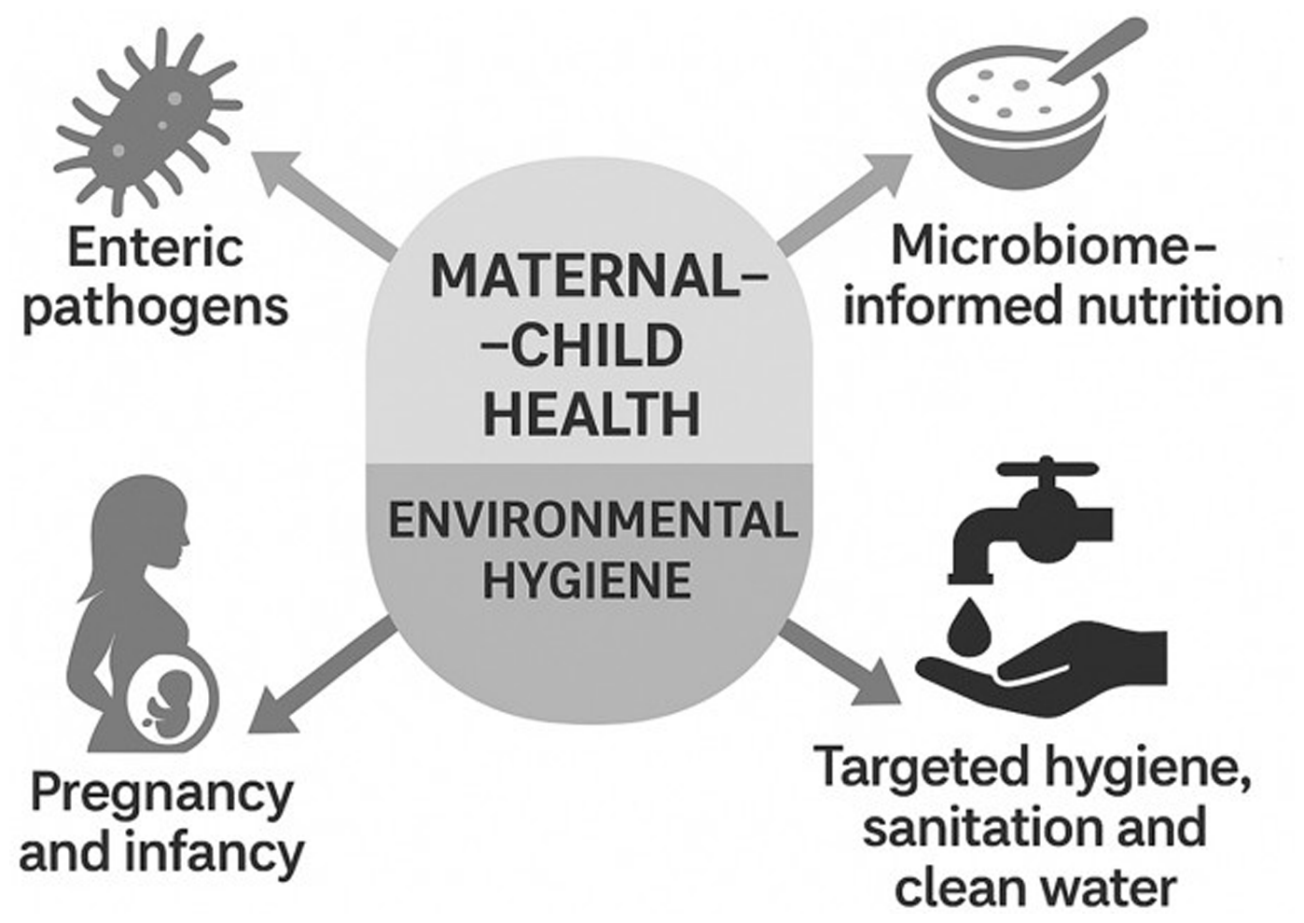
Conceptual framework linking maternal–child health and environmental hygiene (baby WASH) to disruption of the synergistic cycle of malnutrition, immune dysfunction, recurrent enteric pathogen exposure, and EE/EED. Interventions are organized around four operational levers (Box 1): (i) Maternal–child health during the first 1,000 days (preconception, pregnancy, lactation, infancy) to reduce pathogen susceptibility; (ii) environmental hygiene creating pathogen-reduction in living/feeding/play spaces, and reducing fecal-oral exposure from infected people, animals, and fomites including targeted hygiene intervention and hand hygiene; (iii) microbiome-informed nutrition (e.g., prebiotics/probiotics and complementary foods to restore microbial balance and barrier function); and (iv) sanitation and safe water to lower community pathogen loads. Together, these measures aim to reduce pathogen exposure, restore immune homeostasis, improve oral vaccine performance, and enhance linear growth and neurocognitive outcomes in LMICs.

Interrupting the synergistic malnutrition and infection cycle is both an urgently needed public health priority and a foundation for equitable child development. A ‘‘maternal and child health-led, environmental hygiene-first” framework, integrated with microbiome-informed nutrition and sustained behaviour change, may provide a pragmatic path to measurable, durable success in LMICs. With coordinated international support and national policy alignment, LMICs may be able to achieve healthier populations and unlock the human capital that drives long-term economic prosperity (see Figure 10).

## 5 Conclusion

Childhood stunting, manifested by impaired linear growth and neurocognitive development, remains inadequately addressed by currently employed pharmaceutical and non-pharmaceutical interventions. Converging evidence indicates that chronic malnutrition and recurrent enteric infections act synergistically to drive EE/EED, interfere with immune homeostasis, diminish oral vaccine responsiveness, and impair nutrient absorption. These biological effects are compounded by epigenetic remodeling (DNA methylation, histone modifications, and non-coding RNA regulation) established during critical developmental windows, especially the first 1,000 days of life.

To disrupt this synergistic malnutrition and infection cycle, we advocate a maternal- and child- health-centered, environmental hygiene-anchored strategy comprised of the operational elements displayed in Box 1.

BOX 1 **Operational levers proposed for tackling the vicious synergistic cycle of malnutrition, immune dysfunction, and enteric infection**

• Maternal–child health integration (prenatal through early childhood)

Embed pathogen exposure reduction and nutrition support across the continuum of care, from preconception and pregnancy through delivery and the first 2 years. Priorities include antenatal nutrition and micronutrient support, safe birth environments, lactation support, and early life growth monitoring linked to prompt infection prevention and treatment.

Integrate SEEM growth indicators (HAZ, WHZ, WAZ) with longitudinal mapping of DNA methylation (e.g., IGF2, INSR), histone markers (H3K4me3), and microRNAs in enterocytes and immune cells to identify targets for microbiome-directed nutrition, methyl-donor supplementation, and immunomodulatory interventions. This approach should strengthen immune competence and reduce the pathogen susceptibility that generates EE/EED and vaccine underperformance.

• Environmental hygiene (baby WASH) as the operational cornerstone

Create hygienically clean living, feeding, and play spaces that minimize fecal-oral exposures (including animal waste, contaminated soil, toys, and environmental surfaces). Practical components include targeted surface decontamination with broad-spectrum microbicides, safe water and infant food hygiene, vector- and animal-feces control, and consistent hand hygiene. Extending hygiene protections to ‘maternal environments during gestation’ is essential to reduce antenatal pathogen exposure and downstream EE/EED risk.

• Microbiome-informed nutrition and therapeutics

Complement environmental hygiene with microbiome-directed strategies (e.g., prebiotics, probiotics, and complementary foods) designed to restore a healthy microbiome to enhance intestinal barrier function, support immune homeostasis, and improve oral vaccine immunogenicity. Where appropriate, pair these with evidence-based therapeutics for enteric pathogens and mucosal repair.

• Behavioural and Systems Change to Sustain Impact

Structural WASH upgrades must be coupled with context-specific behaviour-change programs, caregiver education, and community engagement to embed daily practices that limit pathogen exposure. Without this integration, infrastructure alone will be undermined by entrenched behaviours in households, childcare settings, and schools including play areas.

## Data Availability

The original contributions presented in the study are included in the article/Supplementary material, further inquiries can be directed to the corresponding author.
