# Supplementary material for: Disrupting the vicious cycle of malnutrition and enteropathy: advancing strategies to improve child physical and neurocognitive outcomes
Source: Front Public Health. 2026 Sep 1;14:1792466. doi: 10.3389/fpubh.2026.1792466 (PMC13575968; doi:10.3389/fpubh.2026.1792466)
Supplement: Supplementary file 1 [file Table_1.DOCX]

**Supplementary Materials**

**Disrupting the Vicious Cycle of Malnutrition and Enteropathy: Advancing Strategies to Improve Child Physical and Neurocognitive Outcomes**

Search strings used for literature search

**Table S1. Search strings applied to Embase**

| **Search** | **Search String** | **Search Results** |
| --- | --- | --- |
| Search 1 | (malnutrition/exp OR 'deficient nutrition' OR 'malnourishment' OR 'malnutrition' OR 'severe acute malnutrition' OR 'underfeeding' OR 'undernourishment' OR 'undernutrition') AND (infection/exp OR 'accidental infection' OR 'acute infection' OR 'autoinfection' OR 'bacterial infections and mycoses' OR 'bacteroid infection' OR 'chain of infection' OR 'infection' OR 'infection mechanism' OR 'infection route' OR 'infections' OR 'infectious disease' OR 'infectivity' OR 'route of infection') AND ((hygiene/exp OR 'hygiene' OR 'hygiene care' OR 'hygiene institute' OR 'regimen sanitatis' OR 'sanitary condition' OR 'skin cleansing') AND ('surface property'/exp OR 'capillarity' OR 'capillary action' OR 'surface' OR 'surface properties' OR 'surface property')) | 37 |
| Search 2 | (malnutrition/exp OR 'deficient nutrition' OR 'malnourishment' OR 'malnutrition' OR 'severe acute malnutrition' OR 'underfeeding' OR 'undernourishment' OR 'undernutrition') AND (infection/exp OR 'accidental infection' OR 'acute infection' OR 'autoinfection' OR 'bacterial infections and mycoses' OR 'bacteroid infection' OR 'chain of infection' OR 'infection' OR 'infection mechanism' OR 'infection route' OR 'infections' OR 'infectious disease' OR 'infectivity' OR 'route of infection') AND ('hand washing'/exp OR 'hand cleansing' OR 'hand drying' OR 'hand hygiene' OR 'hand sanitation' OR 'hand sanitization' OR 'hand scrubbing' OR 'hand washing' OR 'handscrubbing' OR 'handwashing' OR 'scrubbing, hand' OR 'surgical scrubbing' OR 'washing, hand') | 242 |
| Search 3 | (malnutrition/exp OR 'deficient nutrition' OR 'malnourishment' OR 'malnutrition' OR 'severe acute malnutrition' OR 'underfeeding' OR 'undernourishment' OR 'undernutrition') AND (hygiene/exp OR 'hygiene' OR 'hygiene care' OR 'hygiene institute' OR 'hygiene, veterinary' OR 'hygienic assessment' OR 'military hygiene' OR 'regimen sanitatis' OR 'sanitary condition' OR 'skin cleansing') AND ('physical development'/exp OR 'body development' OR 'development, physical' OR 'physical development' OR 'somatic development') | 23 |
| Search 4a | (malnutrition/exp OR 'deficient nutrition' OR 'malnourishment' OR 'malnutrition' OR 'severe acute malnutrition' OR 'underfeeding' OR 'undernourishment' OR 'undernutrition') AND (hygiene/exp OR 'hygiene' OR 'hygiene care' OR 'hygiene institute' OR 'hygiene, veterinary' OR 'hygienic assessment' OR 'military hygiene' OR 'regimen sanitatis' OR 'sanitary condition' OR 'skin cleansing') AND 'environmental enteropathy'/exp | 7 |
| Search 4b (variant on term 3) | (malnutrition/exp OR 'deficient nutrition' OR 'malnourishment' OR 'malnutrition' OR 'severe acute malnutrition' OR 'underfeeding' OR 'undernourishment' OR 'undernutrition') AND (hygiene/exp OR 'hygiene' OR 'hygiene care' OR 'hygiene institute' OR 'hygiene, veterinary' OR 'hygienic assessment' OR 'military hygiene' OR 'regimen sanitatis' OR 'sanitary condition' OR 'skin cleansing') AND 'environmental enteric dysfunction'/exp | 13 |
| Search 5a | (malnutrition/exp OR 'deficient nutrition' OR 'malnourishment' OR 'malnutrition' OR 'severe acute malnutrition' OR 'underfeeding' OR 'undernourishment' OR 'undernutrition') AND ((hygiene/exp OR 'hygiene' OR 'hygiene care' OR 'hygiene institute' OR 'hygiene, veterinary' OR 'hygienic assessment' OR 'military hygiene' OR 'regimen sanitatis' OR 'sanitary condition' OR 'skin cleansing') OR ('hand washing'/exp OR 'hand cleansing' OR 'hand drying' OR 'hand hygiene' OR 'hand sanitation' OR 'hand sanitization' OR 'hand scrubbing' OR 'hand washing' OR 'handscrubbing' OR 'handwashing' OR 'scrubbing, hand' OR 'surgical scrubbing' OR 'washing, hand') OR 'surface hydrophobicity'/exp OR (sanitation/exp OR 'drainage, sanitary' OR 'public facilities' OR 'sanitary drainage' OR 'sanitary service' OR 'sanitation' OR 'toilet facilities' OR 'water softening')) AND ('cognitive development'/exp OR 'cognitive development' OR 'cognitive growth' OR 'development, cognitive') AND ('diet supplementation'/exp OR 'diet supplementation' OR 'dietary supplementation' OR 'nutritional supplementation') | 6 |
| Search 5b  (without nutrition supplements) | (malnutrition/exp OR 'deficient nutrition' OR 'malnourishment' OR 'malnutrition' OR 'severe acute malnutrition' OR 'underfeeding' OR 'undernourishment' OR 'undernutrition') AND ((hygiene/exp OR 'hygiene' OR 'hygiene care' OR 'hygiene institute' OR 'hygiene, veterinary' OR 'hygienic assessment' OR 'military hygiene' OR 'regimen sanitatis' OR 'sanitary condition' OR 'skin cleansing') OR ('hand washing'/exp OR 'hand cleansing' OR 'hand drying' OR 'hand hygiene' OR 'hand sanitation' OR 'hand sanitization' OR 'hand scrubbing' OR 'hand washing' OR 'handscrubbing' OR 'handwashing' OR 'scrubbing, hand' OR 'surgical scrubbing' OR 'washing, hand') OR 'surface hydrophobicity'/exp OR (sanitation/exp OR 'drainage, sanitary' OR 'public facilities' OR 'sanitary drainage' OR 'sanitary service' OR 'sanitation' OR 'toilet facilities' OR 'water softening')) AND ('cognitive development'/exp OR 'cognitive development' OR 'cognitive growth' OR 'development, cognitive') | 93 |
| Additional Search A | ('intestine infection'/exp OR 'enteric infection' OR 'infection, intestine' OR 'intestinal diseases, parasitic' OR 'intestinal infection' OR 'intestine infection' OR 'parasitic intestinal diseases') AND ('cognitive development'/exp OR 'cognitive development' OR 'cognitive growth' OR 'development, cognitive') AND 'hygiene'/exp | 2 |
| Additional Search B | (('intestine infection'/exp OR 'enteric infection' OR 'infection, intestine' OR 'intestinal diseases, parasitic' OR 'intestinal infection' OR 'intestine infection' OR 'parasitic intestinal diseases') OR (malnutrition/exp OR 'deficient nutrition' OR 'malnourishment' OR 'malnutrition' OR 'severe acute malnutrition' OR 'underfeeding' OR 'undernourishment' OR 'undernutrition')) AND ('cognitive development'/exp OR 'cognitive development' OR 'cognitive growth' OR 'development, cognitive') AND ((sanitation/exp OR 'drainage, sanitary' OR 'public facilities' OR 'sanitary drainage' OR 'sanitary service' OR 'sanitation' OR 'toilet facilities' OR 'water softening') OR (hygiene/exp OR 'hygiene' OR 'hygiene care' OR 'hygiene institute' OR 'hygiene, veterinary' OR 'hygienic assessment' OR 'military hygiene' OR 'regimen sanitatis' OR 'sanitary condition' OR 'skin cleansing')) AND (therapy/exp OR 'combination therapy' OR 'disease therapy' OR 'disease treatment' OR 'diseases treatment' OR 'disorder treatment' OR 'disorders treatment' OR 'efficacy, therapeutic' OR 'illness treatment' OR 'medical therapy' OR 'medical treatment' OR 'multiple therapy' OR 'polytherapy' OR 'somatotherapy' OR 'therapeutic action' OR 'therapeutic efficacy' OR 'therapeutic trial' OR 'therapeutic trials' OR 'therapeutics' OR 'therapy' OR 'therapy, medical' OR 'treatment effectiveness' OR 'treatment efficacy' OR 'treatment, medical') | 36 |

**Table S2. Search strings applied to PubMed**

| **Search** | **Search String** | **Search Results** |
| --- | --- | --- |
| Search 1 | Malnutrition + infection + surface hygiene | 11 |
| Search 2 | Malnutrition + infection + hand hygiene | 35 |
| Search 3 | Malnutrition + physical development + hygiene | 177 |
| Search 4 | Malnutrition + environmental enteropathy + hygiene | 51 |
| Search 5 | Malnutrition + (cognitive development) + (((hygiene OR ((surface OR hand hygiene)) OR sanitation))) +(nutritional supplement) | 5 |
| Additional Search A | malnutrition AND (cognitive OR physical development) AND mitigation AND hygiene | 4 |

**Table S3. Search strings applied to Google Scholar**

Variations on the search terms produced 8 possibly useful articles

**Table S4. Search strings applied to SciVal**

**Contents**

Overview report (PDF)

Articles found (abstracts not available from SciVal unfortunately)

Author listing (weighted by number of papers in this topic area)

**Table S5. Other sources**

| **Article Title** | **URL** |
| --- | --- |
| Exploring the role of environmental enteropathy in malnutrition, infant development and oral vaccine response | https://royalsocietypublishing.org/doi/10.1098/rstb.2014.0143 |
| Improving Child Development: A New CDC Handwashing Study Shows Promising Results | <https://www.cdc.gov/handwashing/child-development.html> |
| Hygiene and other early childhood influences on the subsequent function of the immune system | <https://www.sciencedirect.com/science/article/pii/S0006899314004818> |
| Sanitation, Hygiene and Environmental Cleanliness for Child Development | https://mspgh.unimelb.edu.au/__data/assets/pdf_file/0009/3960567/Frontiers19_HealthChildDevelopment-FINAL.pdf |
